# A Unique Virulence Gene Occupies a Principal Position in Immune Evasion by the Malaria Parasite *Plasmodium falciparum*

**DOI:** 10.1371/journal.pgen.1005234

**Published:** 2015-05-19

**Authors:** Uchechi E. Ukaegbu, Xu Zhang, Adina R. Heinberg, Mamadou Wele, Qijun Chen, Kirk W. Deitsch

**Affiliations:** 1 Department of Microbiology and Immunology, Weill Medical College of Cornell University, New York, New York, United States of America; 2 Key Laboratory of Zoonosis, College of Veterinary Medicine, Jilin University, Xi An Da Lu, Changchun, China; 3 University of Sciences Techniques and Technologies of Bamako, Bamako, Mali; Karolinska Institutet, SWEDEN

## Abstract

Mutually exclusive gene expression, whereby only one member of a multi-gene family is selected for activation, is used by the malaria parasite *Plasmodium falciparum* to escape the human immune system and perpetuate long-term, chronic infections. A family of genes called *var* encodes the chief antigenic and virulence determinant of *P*. *falciparum* malaria. *var* genes are transcribed in a mutually exclusive manner, with switching between active genes resulting in antigenic variation. While recent work has shed considerable light on the epigenetic basis for *var* gene activation and silencing, how switching is controlled remains a mystery. In particular, switching seems not to be random, but instead appears to be coordinated to result in timely activation of individual genes leading to sequential waves of antigenically distinct parasite populations. The molecular basis for this apparent coordination is unknown. Here we show that *var2csa*, an unusual and highly conserved *var* gene, occupies a unique position within the *var* gene switching hierarchy. Induction of switching through the destabilization of *var* specific chromatin using both genetic and chemical methods repeatedly led to the rapid and exclusive activation of *var2csa*. Additional experiments demonstrated that these represent “true” switching events and not simply de-silencing of the *var2csa* promoter, and that activation is limited to the unique locus on chromosome 12. Combined with translational repression of *var2csa* transcripts, frequent “default” switching to this locus and detection of *var2csa* untranslated transcripts in non-pregnant individuals, these data suggest that *var2csa* could play a central role in coordinating switching, fulfilling a prediction made by mathematical models derived from population switching patterns. These studies provide the first insights into the mechanisms by which *var* gene switching is coordinated as well as an example of how a pharmacological agent can disrupt antigenic variation in *Plasmodium falciparum*.

## Author Summary

Many eukaryotic pathogens avoid the immune system of their hosts by switching expression between genes encoding their exposed surface antigens. This process, called antigenic variation, is key to the ability of these organisms to cause long-term, chronic infections and represents a major virulence determinant for many infectious diseases. The human malaria parasite *Plasmodium falciparum* achieves this by varying expression between members of the multi-copy *var* gene family. While significant progress has been made in recent years to identify factors that determine whether an individual *var* gene is active or silent, how switching between members of the family is coordinated remains a mystery. Previous work has shown that switching is not random, but rather is coordinated to result in rising and falling populations of parasites expressing single surface antigens. Here we provide evidence that one specific, highly conserved *var* gene occupies a unique position within the switching network, fulfilling a prediction made by mathematical models derived from population switching patterns. These data provide the first insights into the regulatory network underlying antigenic variation by malaria parasites.

## Introduction

Mutually exclusive gene expression, whether selective expression of either the maternal or paternal allele in a diploid organism, the inactivation of an entire sex chromosome during dosage compensation, or the expression of a single gene while silencing all others within a multi-copy gene family, remains one of the most intriguing phenomena in the study of gene expression. Maintaining gene exclusivity is obligatory for proper function of several systems including the regulation of mating-type switching in fission and budding yeast [1], the decision between classes in immunoglobulin switching [2], odorant receptor discrimination within the olfactory system in both vertebrate and invertebrate animals [3], and in X-chromosome inactivation during female mammalian development [4]. Although the intrinsic mechanisms that govern choice, activation and silencing differ remarkably in each system, epigenetic regulation appears to consistently play a part in maintaining mutual exclusivity.

The maintenance of mutually exclusive gene expression is also a key component of the process of antigenic variation employed by many pathogenic organisms that cause chronic, persistent infections [5]. Eukaryotic pathogens including Giardia [6,7], Babesia [8], African trypanosomes [9], and malaria parasites [10] all avoid clearance by the antibody response of their mammalian hosts by continuously altering the surface antigens exposed to the immune system. The genomes of these organisms contain large, multi-copy gene families with each family member encoding a variable form of the surface antigen. Mutually exclusive expression of these genes ensures that only a small portion of the parasites’ repertoire of potential surface antigens is exposed at any time, thereby limiting the infected host’s ability to generate an effective antibody response. Further, by continuously switching which gene is expressed, the parasites can avoid antibodies made earlier in the infection or during previous infections. Thus unlike most examples of mutually exclusive expression from higher eukaryotes in which choice of the active gene is part of terminal differentiation and therefore permanent, for the gene families involved in antigenic variation, activation and silencing are reversible, thereby adding an additional level of complexity to the regulatory system. For switching to occur, a new gene must be chosen for activation while the previously active gene must be simultaneously silenced, thus requiring a mechanism of coordination between different members of the family. In addition, in many instances the switching process does not appear to be entirely random, resulting in populations of millions of individual cells that rise and fall in waves of parasitemia over the course of an infection [11]. The molecular mechanisms that control both activation and silencing of individual genes as well as coordination of the switching process remain virtually entirely undefined.

Antigenic variation mediated by mutually exclusive gene expression is a key component of the lifestyle employed by the causative agent responsible for the most severe form of human malaria, the protozoan parasite *Plasmodium falciparum*. *P*. *falciparum* has a multi-gene family called *var* that encodes the primary antigenic determinant displayed on the surface of infected erythrocytes, a protein called *P*. *falciparum* erythrocyte membrane protein-1 (PfEMP1) [12–14]. The parasite inserts PfEMP1 into the membrane of the infected erythrocyte during asexual development, thereby extended the hyper-variable portion of the protein into the extracellular space where it interacts with a broad range of host cell receptors displayed on the endothelium of blood vessels, including EPCR [15], CD-36 [16] and the placental specific ligand chrondroitin sulfate A (CSA) [17]. The interactions between PfEMP1 and these receptors enable the parasites to tightly adhere and sequester within the deep tissue capillary beds thereby avoiding the peripheral circulation and destruction by the spleen. The genome of *P*. *falciparum* possesses 60 *var* genes interspersed throughout thirteen of the fourteen chromosomes. They are primarily found within the sub-telomeric regions, but approximately a third of the family resides within the central regions of the chromosomes, generally as tandemly arranged arrays. Expression of the *var* gene family operates in a mutually exclusive manner such that only one gene expresses PfEMP1 over many cycles of asexual replication and the other 59 remain transcriptionally silent, thereby avoiding exposure of the encoded proteins to the host’s immune system [18]. Individuals with a healthy immune system can mount a robust antibody response against the surface-exposed PfEMP1, however through expression switching, sub-populations of parasites arise expressing a different *var* gene, resulting in antigenic variation and a persistent infection typified by successive waves of parasitemia.

Significant progress has been achieved in recent years in understanding many aspects of the molecular basis of *var* gene activation and silencing. Changes in the transcription state of a *var* gene do not appear to involve any alterations at the DNA sequence level or to require changes in the presence or absence of typical transcription factors. Thus the process meets the classical definition of an epigenetic mechanism. Silent *var* genes are found in a state of condensed chromatin and known components of heterochromatin appear to be necessary for maintenance of *var* gene silencing [19–22]. Histone marks generally associated with active or silent chromatin in higher eukaryotes are similarly distributed at active or silent *var* genes, however two marks, H3K9me3 and H3K36me3, appear to play particularly prominent roles in *var* gene regulation [23,24]. The silencing mark H3K9me3 seems to be devoted specifically to regulating genes that display variable expression, for example those involved in antigenic variation, alternative invasion pathways and differentiation into the sexual forms. Therefore rather than being found at many transcriptionally silent regions of the genome, it is distributed within the narrow regions of the genome where these genes reside [22,25]. Similarly, the mark H3K36me3 is found primarily at the multi-copy gene families that display mutually exclusivity, however unlike H3K9me3, this mark is found at both active and silent genes [26,27]. Characterization of these marks and their distribution within members of the *var* gene family represents an important step in gaining an understanding of how this gene family is regulated. However, the mechanism underlying expression switching and thus how the individual members of each family are integrated into a single, coordinated unit, remains a mystery.

Although the mechanism by which *P*. *falciparum* is able to maintain the mutual exclusive nature of the *var* multi-copy family remains an enigma in the malaria field, several lines of evidence suggest that switching is not random and that a hierarchy exists in the choice of which genes become activated in the event of a switch. Several studies have shown that different *var* genes have different intrinsic switching rates [28–30]. For example, once activated *var* genes located in central chromosomal regions are relatively stable and rarely switch in the absence of selection. In contrast, those in the subtelomeric regions of the chromosomes tend to switch much more frequently, resulting in higher “on” and “off” rates. Careful measurement of *var* gene switching dynamics observed in clonal parasite populations grown for several months in culture revealed a surprisingly simple switching pattern in which an initial dominant *var* transcript was replaced over time by one or a small number of alternative members of the family [29]. Mathematical modeling of the observed in vitro switching events, as well as population dynamics observed in experimental infections, suggested that these parasite populations were highly unlikely to be undergoing random switching. Rather, two separate mathematical models predicted that *var* gene switching occurs through the use of transiently activated genes that act as “switch intermediates”, thereby adding a level of uniformity and coordination to the switching process [29]. However the identity of such intermediates was not defined.

In an unrelated study, clonal parasite cultures were grown without selection for over ~ 200 generations (>1 year) and found to display a very high activation rate of a particular gene within the family called *var2csa*, leading the authors to suggest that expression of *var2csa* might represent a “default” state [31]. *var2csa* is a unique member of the *var* family that encodes a form of PfEMP1 that binds exclusively to chondroitin sulfate A (CSA) displayed on the surface of synciciotrophoblasts in the placenta [32]. Unlike most *var* genes, *var2csa* appears to be universally conserved among parasite isolates from all over the world, suggesting strong selection pressure to maintain this gene within the parasite’s genome [33]. This is surprising considering that the PfEMP1 encoded by this gene appears to be useful only when expressed in women during their first pregnancy, a relatively small portion of the human population. Further, this particular *var* gene is unique amongst members of the family in that it is also translationally repressed and thus frequently does not express PfEMP1 even when transcriptionally activated [31,34,35]. Even more intriguing, transcripts from this gene are frequently detected in non-pregnant individuals [36,37], an observation that is puzzling given the presumed role of the gene in placental binding. These observations suggest that this gene might have an alternative function beyond simply encoding a unique form of PfEMP1.

In previous work, we also observed the specific upregulation of *var2csa* upon manipulation of chromatin marks specific to *var* genes and telomeres [27]. Here, we extend these findings and show that *var2csa* occupies a unique position within the *var* switching network. Specifically, manipulation of histone modifications found within chromatin surrounding *var* genes using both genetic and chemical means results in the transcriptional activation of *var2csa*. Activation of *var2csa* coincides with the simultaneous silencing of other members of the family, indicating that mutually exclusive expression was maintained and thus that the induction of *var2csa* is indeed a “true” switching event. This activation was specific to the conserved *var2csa* locus on chromosome 12 and did not extend to a second copy located at a different chromosomal position within the genome, even though both copies contain virtually identical regulatory domains. This indicates that *var2csa* activation involves coordination at the level of the entire genome rather than simply the sequence dependent recruitment of transcriptional activators to the UpsE-type regulatory region. These data suggest that *var2csa* displays many of the characteristics of a previously predicted switch intermediate and that its function likely extends well beyond simply encoding a conserved form of PfEMP1. In addition, this work provides clues as to how overall *var* gene switching events are coordinated as well as the first example of a chemical method to manipulate *var* gene expression patterns.

## Results

### Disruption of PfSET2 recruitment by RNA pol II induces activation of *var2csa*

**Fig 1:**
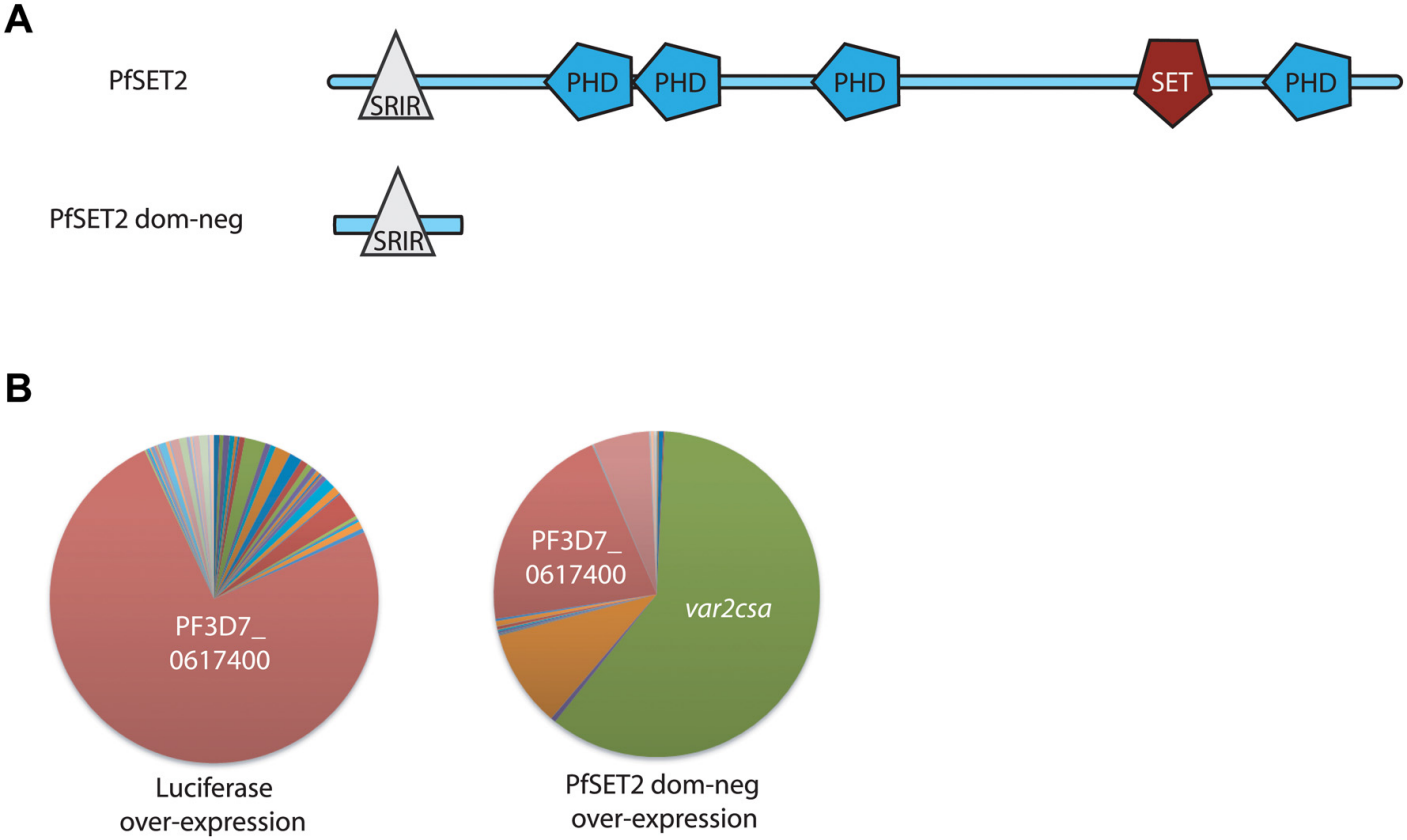
Effect of over-expression of PfSET2 dom-neg on *var* gene expression. (A) Schematic diagram of the domain structure of PfSET2. The top image shows the conserved domains identified using SMART (Simple Modular Architecture Research Tool, http://smart.embl-heidelberg.de). PHD domains are shown as blue polygons while the methyltransferase domain (labeled SET) is shown in red. The SET2 Rpb1 Interacting Region (SRIR) is shown as a white triangle. (B) *var* gene expression is shown as a pie-chart, with each slice of the pie representing the fraction of the total *var* mRNA pool transcribed from each *var* gene. The left chart shows the *var* gene expression pattern in a population over-expressing firefly luciferase. This is unchanged from the untransfected population and the annotation number of the dominant gene is shown in white text. The chart on the right shows the *var* gene expression pattern in a population after over-expression of the PfSET2 dominant negative construct for 6 weeks. *var2csa* (PF3D7_1200600, shown in green) has become the dominant transcript. Individual copy number values for each transcript are shown in S1 Fig.

Recent work showed that similar to H3K9me3, the histone mark H3K36me3 is deposited primarily on chromatin associated with multi-copy gene families, including both active and silent *var* genes, but not on the vast majority of other regions of the *P*. *falciparum* genome [26,27]. Consistent with a specific role for the H3K36me3 mark in regulating variant gene expression, knocking out PfSET2, the histone methyltransferase that deposits the H3K36me3 mark, did not have a significant effect on parasite growth but led to leaky expression of all *var* genes, thus implicating a role in maintaining mutually exclusive expression [26]. We previously showed that PfSET2 binds directly to the C-terminal domain (CTD) of RNA polymerase II, providing a mechanism for its recruitment to specific regions of the genome [27]. Using in vitro co-immunoprecipitation assays, we identified the region of PfSET2 that interacts with the CTD (called the SET2-RNA pol II Interaction region or PfSRIR, Fig 1A) [27]. We further validated the function of this domain by creating a “dominant-negative” construct that consisted of only the PfSRIR. We hypothesized that this truncated version of PfSET2 would compete with the endogenous protein for binding to RNA pol II, thereby reducing recruitment without affecting actual enzyme levels. As predicted, over-expression of the PfSET2 dom-neg resulted in profound changes in *var* gene expression patterns [27]. Surprisingly however, in multiple independent transfections, over-expression of the dominant-negative construct did not lead to random *var* gene switching or de-repression of multiple *var* genes, but rather consistently resulted in the specific activation of the single, conserved gene *var2csa*. Over-expression of firefly luciferase using the same methodology had no effect on *var* gene expression. Similar, independent experimental results are shown in Fig 1B.

While most members of the *var* gene family are highly variable when compared between parasite lines [38,39], *var2csa* is conserved among isolates from all over world [33]. It is also unusual in that it has a unique promoter (called UpsE) and is translationally controlled by the presence of a repressive upstream open reading frame within the 5’ leader of its transcript [34,35,40,41]. Additionally, although *var2csa* transcripts appear to only be actively translated into PfEMP1 by parasites infecting first-time pregnant women, *var2csa* transcripts have also been found in isolates retrieved from men and non-pregnant women infected with *P*. *falciparum* [36,37]. The transcripts appear to not be translated in these individuals and their significance is not understood.

To further understand the upregulation of this unusual *var* gene in response to over-expression of the PfSET2 dom-neg, we conducted a more detailed analysis of its activation. In these experiments, parasites were stably transfected with plasmids encoding either the PfSET2 dom-neg or a control plasmid expressing firefly luciferase. For transgene expression, we employed the expression system originally described by Epp et al [42]. This system utilizes a bidirectional promoter to drive expression of both the transgene (either the PfSET2 dom-neg or luciferase) and the drug selectable marker blasticidin-S-deaminase (*bsd*). In cultures of transfected parasites, the copy number of the plasmid can be manipulated by altering the concentration of blasticidin in the culture medium. When blasticidin concentrations are increased, parasites are selected that carry an increased copy number of the plasmid, thereby increasing expression levels of both the *bsd* cassette and the transgene. In this way, expression levels of the transgene can be controlled.

**Fig 2:**
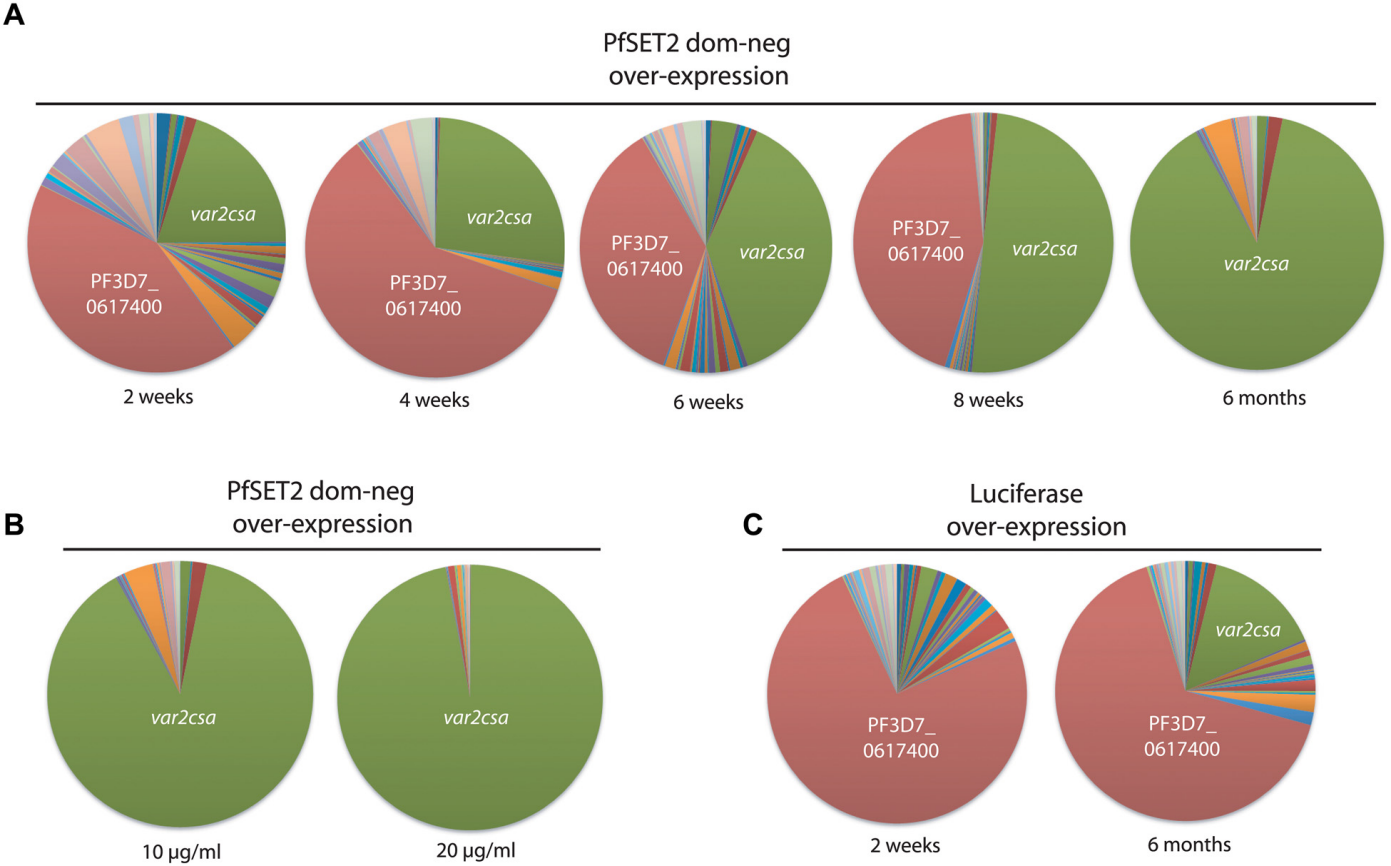
Dynamics of *var2csa* activation in response to PfSET2 dom-neg over-expression. (A) *var* gene expression profiles were determined at 2 week intervals after induction of PfSET2 dom-neg over-expression. The total *var* gene transcript pool is represented as a pie chart, with each slice of the pie representing the fraction of the total *var* mRNA pool transcribed from each *var* gene. In this population of parasites, the *var* gene PF3D7_0617400 (shown in red) was initially dominant, however over time it is replaced by *var2csa* (shown in green). (B) The *var* gene pattern in response to two different levels of PfSET2 dom-neg over-expression. Increasing the selection pressure from 10 μg/ml to 20 μg/ml results in even greater dominance of the *var2csa* message as a portion of the total *var* mRNA pool. (C) In response to luciferase over-expression, the *var* gene pattern does not change substantially over 6 months, although activation of *var2csa* is detectable. Individual copy number values for each transcript are shown in S2, S3 and S4 Figs.

As previously reported, when grown under low blasticidin concentrations (2 μg/ml), expression levels of the transgene are low, and we detect little change in *var* gene expression patterns in parasites transfected with either construct. However growth under high blasticidin concentration (10 μg/ml) selected for increased plasmid copy number, high levels of transgene expression and induction of *var2csa* expression in the parasites over-expressing the PfSET2 dom-neg (Fig 1B and [27]). To investigate how quickly *var2csa* activation occurs, we sampled cultures every 2 weeks after shifting the cultures from 2 μg/ml to 10 μg/ml and determined the *var* gene expression profile by Q-RT-PCR using the method originally described by Salanti et al. [43]. A representative time course of activation is shown in Fig 2A. Induction of *var2csa* was easily detectable as early as 2 weeks, becoming second in abundance to only the previously dominant transcript. *var2csa* consistently became the dominant *var* gene after six weeks of drug pressure although the *var* gene that was initially dominant was still expressed at relatively high levels within the population. Continued monitoring of the *var* gene expression pattern for an additional three months showed that the initial dominant *var* gene was no longer strongly expressed and that *var2csa* had become the only highly expressed *var* gene in this population of parasites. The dominance of *var2csa* further increased upon exposing the cultures to even higher concentrations of blasticidin (20 μg/mL) (Fig 2B). In the line over-expressing luciferase, no change in the dominantly expressed *var* gene was detected even after 6 months of selection under 10 μg/ml blasticidin, although *var2csa* expression was eventually detectable in this population (Fig 2C). This lower level of *var2csa* activation is reminiscent of that previously reported by Mok et al. [31]. These results suggest in response to over-expression of the PfSET2 dom-neg, all of the parasites in the culture are switching to *var2csa* at an accelerated rate, resulting in the eventual dominance of this gene in the population. Importantly, there is no selection for VAR2CSA expression at the protein level, thus these switching events appear to represent purely a bias toward transcriptional activation of *var2csa*.

### Activation of *var2csa* by over-expression of the PfSET2 dom-neg is independent of the previously active *var* gene

**Fig 3:**
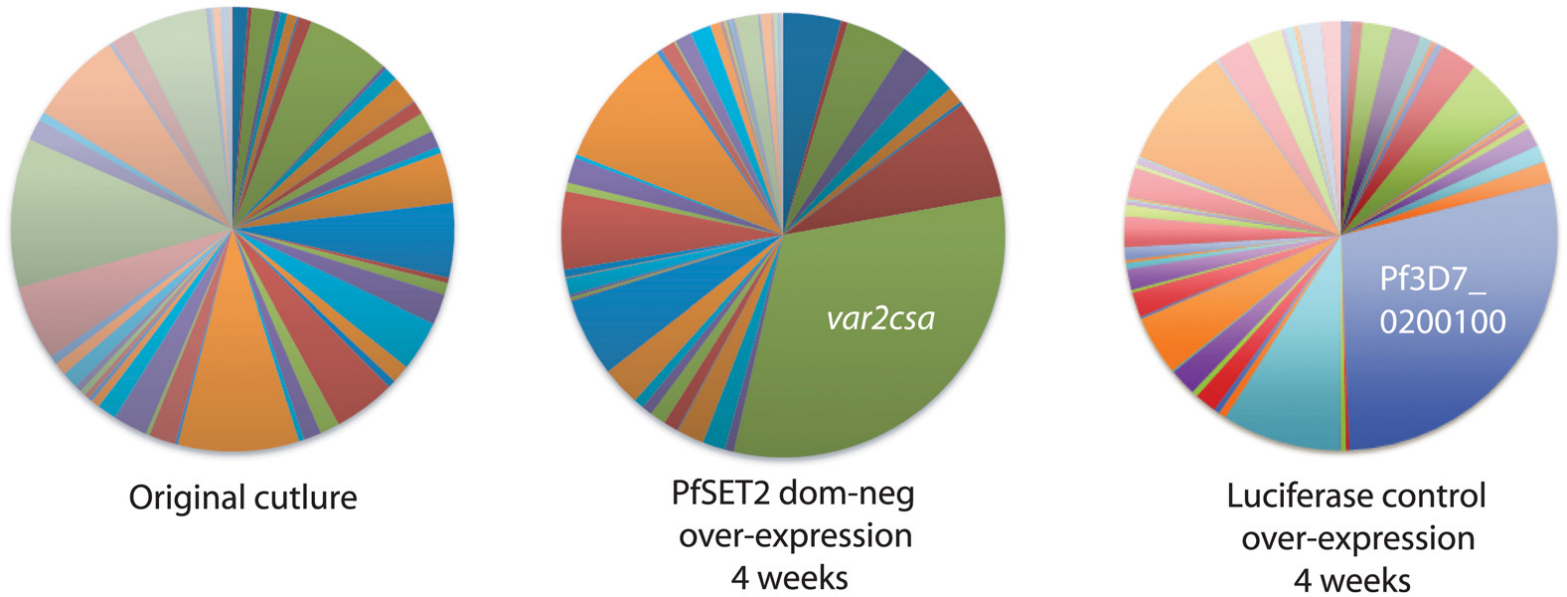
Induction of *var2csa* expression in response to PfSET2 dom-neg over-expression in a heterogenous population expressing many *var* genes. The original population expressed many *var* genes and there was no single gene that was dominant (left panel). After selection with 10 μg/ml blastidicin leading to over-expression of the PfSET2 dom-neg construct for 4 weeks, *var* gene expression began to shift to *var2csa*, resulting in this gene being the most highly expressed gene in the population (middle panel). Similar over-expression of luciferase did not lead to expression of *var2csa* (right panel), while a different *var* gene became somewhat highly expressed, indicating that switching can occur. Individual copy number values for each transcript are shown in S5 Fig.

In our previous experiments, we found that over-expression of the PfSET2 dom-neg construct could induce switching of expression from one *var* gene to *var2csa* within a population that was relatively homogenous at the time the experiment was initiated. However, in the absence of any manipulation, different subclones of parasites frequently display very different switching rates resulting in the activation of different members of the *var* gene family. What influences these different switching dynamics is not clear, although the subtype of the *var* gene that is initially dominant has been proposed to influence switching rates [28,44]. To determine if *var2csa* activation was broadly true for parasites initially expressing any *var* gene, we studied the effect of PfSET2 dom-neg over-expression on populations of parasites that were largely heterogeneous and in which no particular *var* gene was dominantly expressed. Similar to previous experiments, four weeks after initiating PfSET2 dom-neg over-expression *var2csa* expression was clearly induced. In this case, since no other *var* genes were dominant in this population, *var2csa* became the most highly expressed *var* gene after only a month of 10 μg/ml blasticidin treatment (Fig 3). Over-expression of luciferase did not result in switching to *var2csa*, although switching to other *var* genes was evident as has been previously observed [28]. These data suggest that regardless of which *var* gene is initially expressed, parasites exposed to PfSET2 dom-neg over-expression switch with high frequency to expressing *var2csa*.

### Exposure of parasites to a histone methyltransferase small-molecule inhibitor also induces upregulation of *var2csa*

**Fig 4:**
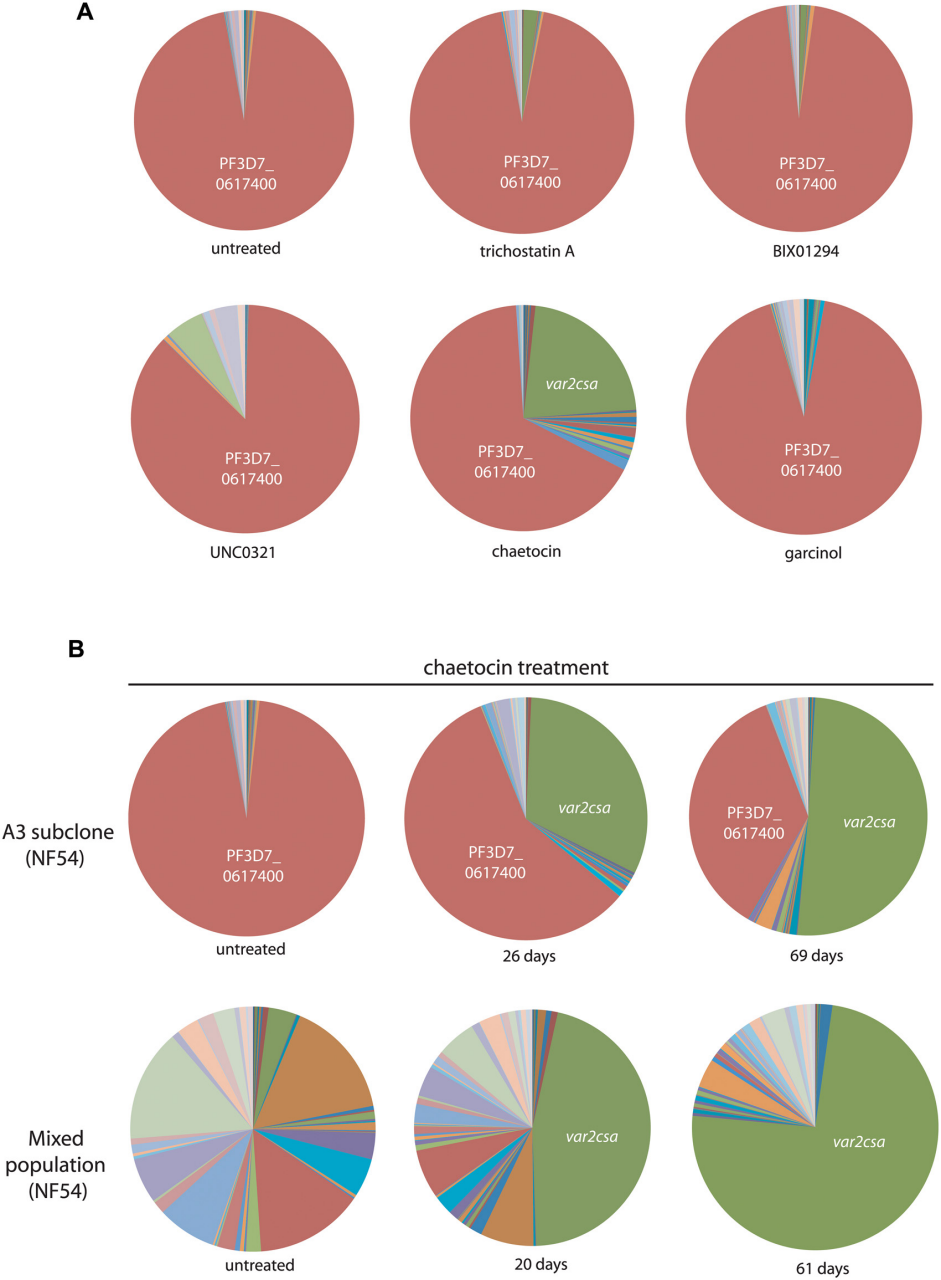
Induction of *var2csa* expression by treatment with a histone methyltransferase inhibitor. (A) The A3 subclone of the NF54 line of *P*. *falciparum* was treated for 2 weeks with sub-IC_50_ concentrations of various compounds known to inhibit histone modifiers in high eukaryotes. After 2 weeks of exposure, the *var* gene expression pattern was determined for each population and shown as a pie chart. In all cases the *var* gene that was originally dominant in this population (PF3D7_0617400, shown in red) remained dominant, however the population exposed to chaetocin displayed a significant proportion that had switched to *var2csa* (green). (B) Two subclones of NF54 (A3 and a mixed population) were cultured in the presence of chaetocin and their *var* gene expression patterns determined after varying lengths of time. In both cases, *var2csa* became the dominant *var* transcript detectable in the population. Individual copy number values for each transcript are shown in S6 Fig.

Our experiments with the PfSET2 dom-neg indicated that genetic manipulation of an epigenetic factor involved in the regulation of *var* genes, specifically recruitment of the enzyme that deposits the H3K36me3 mark, could provide insight into the dynamic nature of transcriptional regulation of the *var* family. Therefore we reasoned that chemical inhibitors known to affect histone modifier activity in other organisms could potentially have a similar impact on *var* gene expression. Our initial experiments involved obtaining IC_50_ values of known inhibitors using a SYBR Green-based parasite viability assay. Two subclones of NF54 were exposed for 72 hours to a series of compounds that included three histone methyltransferase inhibitors—BIX01294, chaetocin and UNC0321—a histone acetyltransferase inhibitor, Garcinol, and the histone deacetylase inhibitor trichostain A (TSA). Chloroquine, a drug known to not have an effect on *var* gene regulation, and parasites not treated with inhibitors, were used as controls. IC50s were determined for all compounds and are shown in Table 1. As an initial screen to investigate any effects the inhibitors had on *var* gene expression, we exposed a clone of NF54 that displays a relative uniform *var* gene expression pattern to two-thirds of the IC_50_ concentrations for 2 weeks and assayed for any changes in *var* gene expression. Following the same procedure as established for the PfSET2 dom-neg experiments, cultures were synchronized for ring stages, RNA isolated and cDNA synthesized. *var* gene expression patterns were then analyzed by Q-RT-PCR. Among all compounds, parasites treated with the histone methyltransferase inhibitor chaetocin showed a nearly identical upregulation of *var2csa* as previously observed after over-expression of the PfSET2 dom-neg (Fig 4A). To further investigate the effect of chaetocin treatment, we conducted a time course of exposure using two subclones of NF54. After approximately six weeks of exposure to chaetocin, *var2csa* became the dominant *var* transcript expressed by the population, with the initial dominant *var* gene having lower transcript levels in both NF54 subclones (Fig 4B).

**Table 1:** Calculated IC_50_ values of BIX-01294, chaetocin, UNC0321, Garcinol, trichostatin A (TSA), and chloroquine (CQ) treated NF54 parasites.

BIX01294	Chaetocin	UNC0321	Garcinol	TSA	CQ
50+/-12 nM	949+/-86 nM	6.5+/-1.5 nM	567+/-43 nM	44+/-4 nM	40+/-14 nM

Chaetocin is a su(var)3–9 methyltransferase inhibitor that inhibits di- and trimethylation of H3K9 in human cells and has been used to study heterochromatin effects on gene expression in numerous cancer studies [45]. Bioinformatic analysis as well as enzymatic assays have identified PfSET3 (PF3D7_0827800) as possessing the highest homology within the su(var)3–9 family in *P*. *falciparum* and displaying the appropriate methyltransferase activity, thus making it the most likely target of chaetocin [46]. It is perhaps not surprising that of all the inhibitors used in our study, the cultures that were treated with chaetocin were the only cultures that displayed changes in *var* gene expression patterns. H3K9me3, the mark targeted by chaetocin, is similar to H3K36me3 in that it is found at limited sections of the genome, in particular *var* genes and telomeric regions [22,25]. By treating with sub-IC50 concentrations, it is likely that PfSET3 activity was partially inhibited rather than being abolished, in a manner similar to the effect of PfSET2 dom-neg over-expression on PfSET2 activity. Thus in both experimental approaches, partial inhibition of the enzymes responsible for the deposition of epigenetic marks specific to variant antigen encoding gene families resulted in induction of *var2csa* expression. These data again suggest that *var2csa* occupies a unique position in the *var* gene switching hierarchy. In both the PfSET2 dom-neg experiments and after chaetocin treatment we did not observe any substantial changes in the quantity of cellular H3K9me3 or H3K36me3 by Western blotting analysis. This is likely because the inhibition of methyltransferase activity is partial and subtle, and in the case of H3K9me3, complete inhibition of H3K9me3 deposition could have detrimental effects on telomere stability and thus parasite viability.

### Induction of *var2csa* by genetic and pharmacological methods is specific to the chromosome 12 locus

Since the experiments involving both PfSET2 dominant-negative over-expression and chaetocin treatment were done in subclones of the NF54 isolate of parasites, we were curious as to whether upregulation of *var2csa* by these two methods could also be detected in an alternative parasite strain, and further whether this effect was specific to the genomic position on chromosome 12. To address these questions, we sought to perform the same genetic and chemical experiments on a parasite line derived from a distinct geographical isolate. For these experiments we chose the Central American isolate HB3. In addition to the availability of the complete sequence of *var2csa*, HB3 is one of a handful of *P*. *falciparum* isolates known to possess multiple but distinct *var2csa* genes at different chromosomal loci [47,48]. Like NF54, HB3 isolates have a *var2csa* locus mapped to the subtelomeric region of chromosome 12, with the second *var2csa* copy located in a subtelomeric region of chromosome 1. Both *var2csa* genes possess similar regulatory regions, including the upstream open reading frame previously shown to act as a translational repressor (Broad Institute *P*. *falciparum* database), and both were shown to be capable of transcriptional activity [47,49]. Thus, at least at the level of DNA sequence, both genes appear to be equivalent. Given these characteristics, HB3 is an interesting isolate to investigate the effects of PfSET2 dom-neg over-expression and chaetocin treatment.

**Fig 5:**
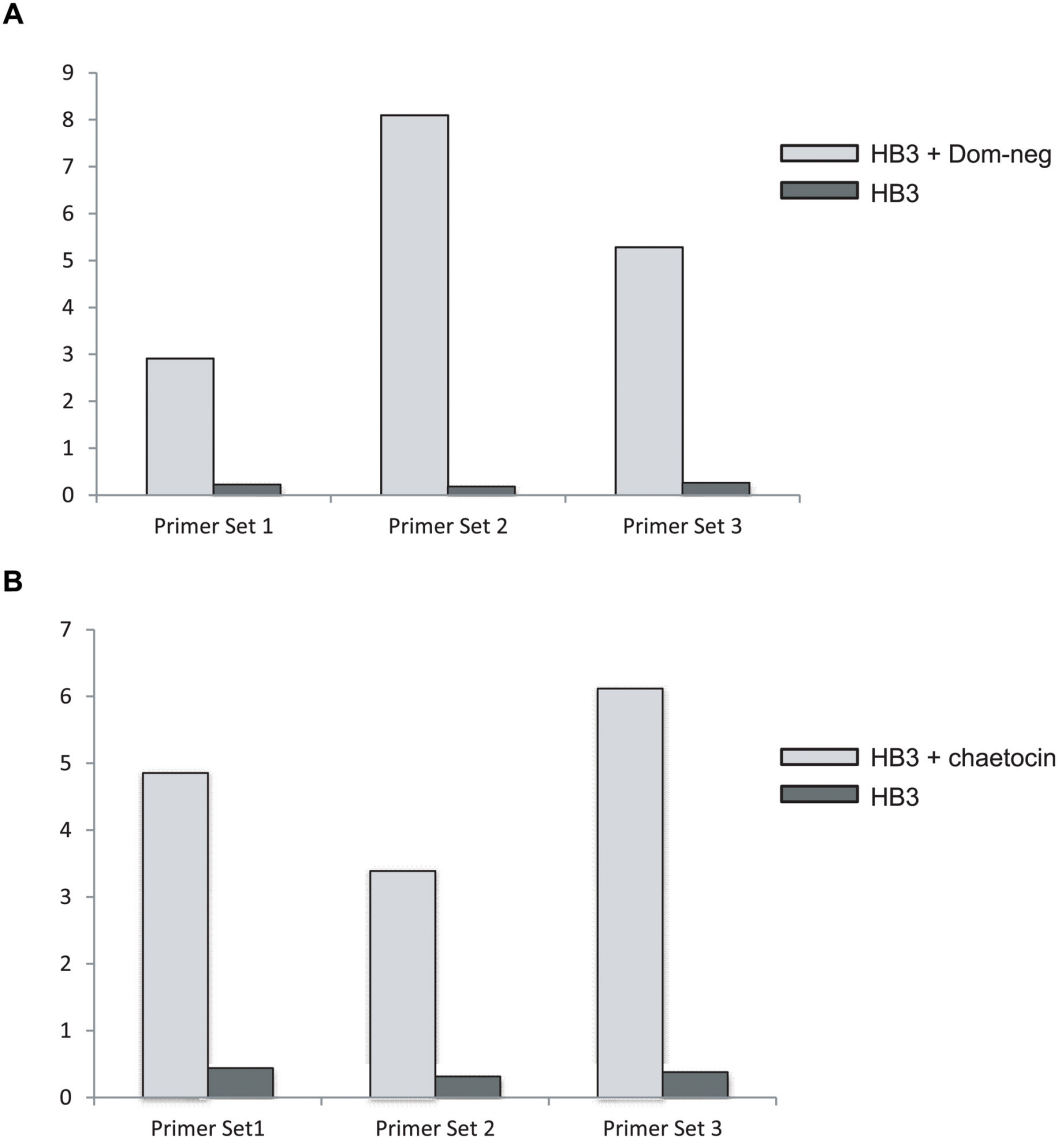
Induction of *var2csa* in the parasite line HB3. The expression level of *var2csa* in HB3 was determined by Q-RT-PCR and displayed as relative copy number as compared to seryl-tRNA synthetase. Three separate PCR primer pairs (shown in Table 2) were used that anneal at different points within the *var2csa* coding region. (A) mRNA expression levels of *var2csa* are shown before and after 3 weeks of exposure to over-expression of the PfSET2 dom-neg expression construct. B. mRNA expression levels of *var2csa* are shown before and after 3 weeks of exposure to chaetocin.

The same PfSET2 dom-neg over-expression and chaetocin treatments that were done in the subclones of NF54 were also carried out in HB3 parasites. Q-RT-PCR analysis indicated that *var2csa* was similarly upregulated in HB3 parasites subjected to these treatments (Fig 5A and 5B). Due to our lack of a complete primer set capable of detecting transcripts from each individual *var* gene within the HB3 genome, we were unable to determine what proportion of the entire *var* mRNA pool had been shifted to *var2csa*. However, all three *var2csa*-specific primers pairs detected a substantial upregulation, ranging from 13 to 40 fold, in response to both over-expression of the PfSET2 dom-neg and chaetocin treatment. Interestingly, in the dominant-negative experiments, only low concentrations of blasticidin (2 μg/mL) were required for *var2csa* upregulation, unlike in the experiments with subclones of NF54 in which *var2csa* activation required selection with 10 μg/ml blasticidin. Strong upregulation of *var2csa* was also observed in HB3 cultures that were treated with chaetocin. Interestingly, high levels of induction of *var2csa* were observed after ~3 weeks of dom-neg over-expression or chaetocin treatment of HB3, a more rapid response than observed with the NF54 subclones. Nevertheless, these data indicate that induction of *var2csa* by both methods is not specific to subclones of NF54.

**Fig 6:**
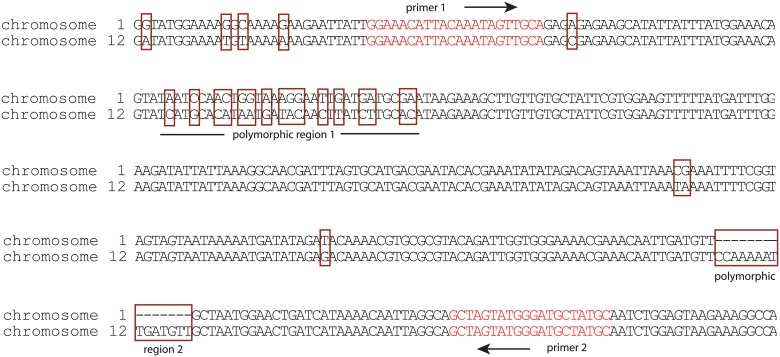
Induction of *var2csa* in HB3 specifically activates the locus on chromosome 12. The nucleotide sequences of the two copies of *var2csa* found within the genome of HB3 are shown. Of note are the polymorphisms (surrounded by red boxes) that easily distinguish the gene found on chromosome 1 from that found on chromosome 12. In particular, two highly polymorphic regions are labeled. The location and sequence of PCR primers (red text) that amplify across these polymorphic regions are shown. Sequencing of this PCR product after induction of *var2csa* expression by chaetocin treatment or over-expression of PfSET2 dom-neg detected only the locus on chromosome 12.

As mentioned above, HB3 is one of a few malaria isolates that have been shown to have more than one copy of *var2csa*. Since we observed that treatment of HB3 parasites yielded a similar phenotype to clones of NF54, we went on to decipher which *var2csa* locus is upregulated in response to manipulation of the H3K36me3 and H3K9me3 marks. We first verified that our HB3 line did indeed have two *var2csa* loci. Southern blots of genomic DNA detected the precise fragments predicted from the available HB3 genome sequence and previously reported by Kraemer et al and Sander et al [47,48] confirming that our HB3 isolate does indeed possess two *var2csa* genes. Using the complete *var2csa* sequences previously reported, we were able to design *var2csa*-specific primers that amplified across a region that contains several single nucleotide polymorphisms as well as 2 regions of extensive sequence differences. These regions could easily distinguish between transcripts from the two *var2csa* copies (Fig 6), thus enabling us to determine which copy is induced by our treatments. PCR amplifications using cDNA from either chaetocin treated parasites or parasites over-expressing the PfSET2 dom-neg yielded single products, and sequencing of both PCR products showed that the amplified bands were specific for the *var2csa* gene located on chromosome 12. It is noteworthy that the conserved *var2csa* copy found within the genomes of all sequenced *P*. *falciparum* lines is located at this position in the genome, suggesting that the gene activation we observe in response to PfSET2 dom-neg over-expression or chaetocin treatment is specific for this conserved locus rather than simply the sequence of the *var2csa* regulatory region. In other words, these treatments do not simply activate the UpsE promoter, but rather induce expression of the specific *var2csa* locus found on chromosome 12. Previous work found that a recombination event that altered the subtelomeric region of chromosome 12 downstream of *var2csa* resulted in persistent activation of this gene, further suggesting that this chromosomal locus possesses additional regulatory capacity [50].

### Activation of *var2csa* is a true switching event and not a result of simply de-silencing the *var2csa* locus

The induction of *var2csa* expression in response to PfSET2 dom-neg over-expression or chaetocin treatment was pronounced and reproducible. While it was apparent that *var2csa* expression was upregulated, it was not clear if this represented simply de-silencing of the *var2csa* locus without switching, infrequent switching to *var2csa* followed by selective outgrowth of those parasites that had switched, or frequent and repeated switching to *var2csa* by most or all parasites within the population. To address these questions, we repeated the chaetocin treatments using a previously derived NF54 line called DC-J in which exon 1 of a *var* gene on chromosome 2 was replaced with the *blasticidin S deaminase (bsd)* selectable marker using double crossover integration [51]. Growth of these parasites under blasticidin pressure results in selection of a population that only expresses the *bsd* containing *var* locus and has silenced the rest of the *var* gene family. If chaetocin treatment simply leads to de-silencing of *var2csa* in the absence of switching, treatment of DC-J with both blasticidin and chaetocin should result in simultaneous expression of both *bsd* and *var2csa*. If chaetocin treatment doesn’t influence switching but rather simply selects for growth of parasites that express *var2csa*, then blasticidin selection should kill parasites that have switched away from the *bsd* encoding *var* locus and prevent outgrowth of *var2csa* expressing parasites. In this case, treatment of DC-J with both blasticidin and chaetocin will not alter *var* gene expression patterns and *bsd* will remain the dominantly expressed *var* gene. Lastly, if chaetocin treatment induces frequent, “true” switching to *var2csa*, parasites that have switched will be killed by blasticidin and thus *var2csa* will not become the dominantly expressed *var* gene. Further, if many or most of the parasites in the culture are induced to switch to *var2csa* in response to chaetocin treatment and consequently are killed by blasticidin, the growth rate of the culture should be severely reduced, and eventually the population should die out. Given the time course experiments shown in Fig 2, alterations in growth rates should be detectable within 6 weeks of first exposure to chaetocin.

**Fig 7:**
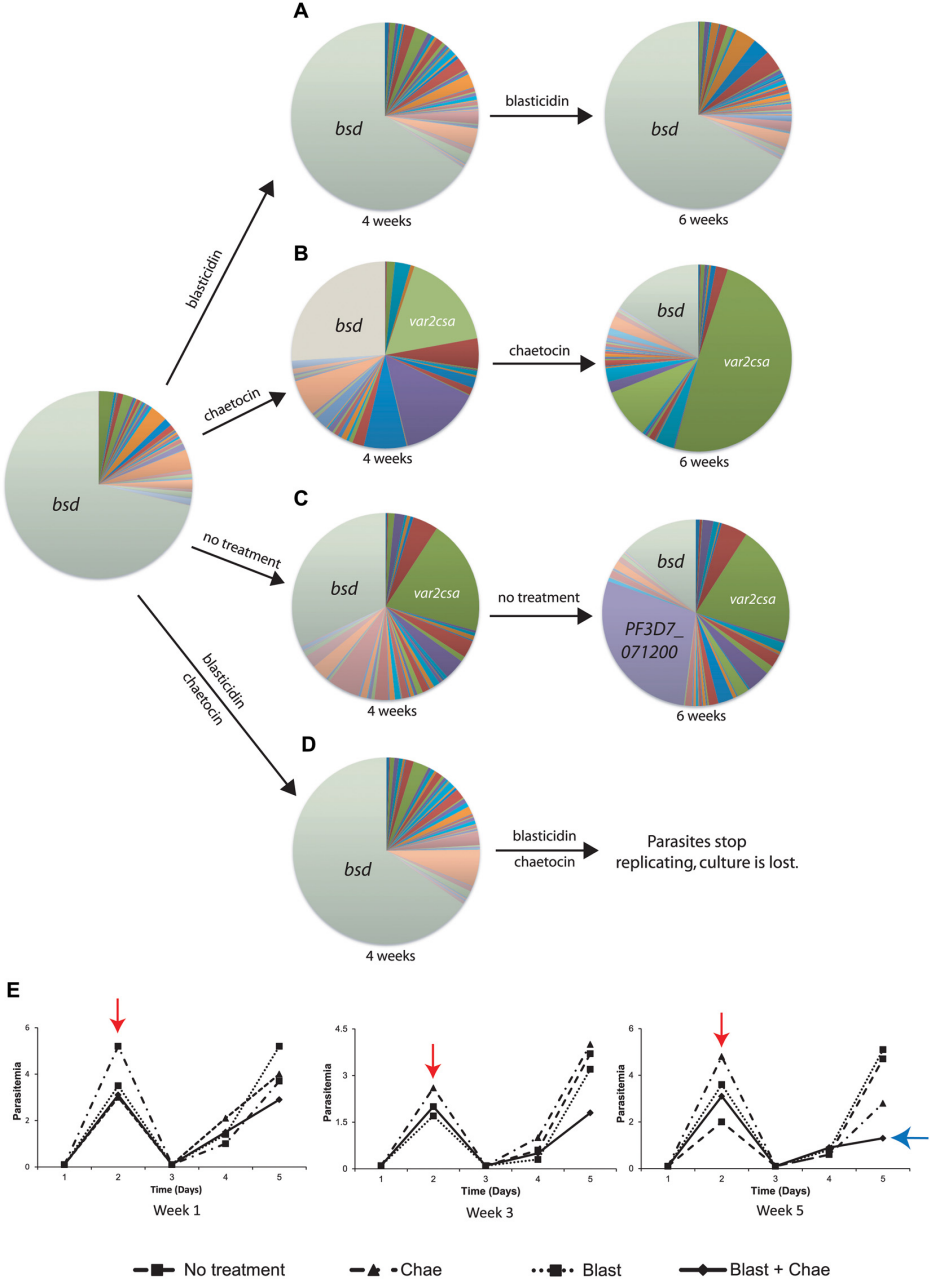
*var2csa* expression in response to chaetocin treatment is a true switching event and maintains mutually exclusive expression. The transgenic parasite line DC-J contains a modified *var* gene that encodes the drug resistance marker *blasticidin-S-deaminase* (*bsd*). Thus upon selection with blasticidin, these parasites are forced to express *bsd* and consequently silence the rest of the *var* gene family. DC-J parasites were grown under different conditions and the *var* gene expression profile determined by Q-RT-PCR and displayed as pie charts. (A) Growth of DC-J in the presence of blasticidin results in stable expression of *bsd* which remains the dominant *var* gene transcript detectable. (B) Growth of DC-J in the absence of blasticidin but in the presence of chaetocin results in induction of *var2csa* expression. *var2csa* has become the dominant *var* gene expressed by this population after 6 weeks of exposure to chaetocin. (C) Growth of DC-J parasites in the absence of blasticidin selection results in switching to several other *var* genes, including *var2csa*. After 6 weeks of growth in the absence of selection, both *var2csa* and a third *var* gene (PF3D7_071200, shown in purple) have become prominently expressed. (D) DC-J grown in the presence of both blasticidin and chaetocin results in stable expression of *bsd* for 4 weeks, after which the population fails to grow and eventually dies out. (E) Parasite growth charts for populations treated with blasticidin, chaetocin, blasticidin + chaetocin or untreated. Parasitemias were calculated daily by blood smear and three weeks are shown. Populations were allowed to replicate until they reached ~3% parasitemia, at which time the population density was reduced to 0.5% (red arrow) and the culture allowed to continue. By the end of week 5, the population exposed to both blasticidin and chaetocin failed to replicate efficiently (blue arrow) and the population died out by week 6. Individual copy number values for each transcript are shown in S7–S11 Figs.

To address these questions, we first verified that the DC-J parasites grown in the presence of blasticidin were indeed exclusively transcribing the *var* locus encoding the *bsd* selectable marker. To assay *var* gene expression patterns, we again utilized Q-RT-PCR using the previously described *var* gene primer set as well as primers specific to *bsd*. As expected, under blasticidin selection the *bsd* containing locus was by far the dominant *var* transcript detected. The culture was then divided into four different flasks for testing different conditions over a total of six weeks. The four cultures consisted of (i) DC-J parasites maintained under blasticidin pressure as control, (ii) chaetocin treated DC-J parasites (iii) DC-J without any treatment to measure the default *var* switching rate, and (iv) DC-J parasites selected with both blasticidin and chaetocin (Fig 7). The *bsd* containing *var* locus remained the dominant transcript in the culture that was maintained under blasticidin selection throughout the duration of the experiment (Fig 7A). Similar to the experiments performed with the NF54 cultures, *var2csa* became the dominant transcript after six weeks of treatment with chaetocin alone (Fig 7B). Interestingly, after a month in culture in the absence of any selection, *var2csa* transcripts were detectably expressed alongside the *bsd* gene in the DC-J parasites that were not undergoing any treatment (Fig 7C). Further analysis after two more weeks in culture detected another prominent gene, thus three genes—*bsd*, *var2csa* and *PF3D7_071200*, were now highly expressed in the population, indicating that in the absence of blasticidin selection, this population is undergoing frequent switching. The detection of *var2csa* transcripts in these cultures is reminiscent of the study performed by *Mok et al* in which they similarly detected frequent switching to *var2csa* expression in unselected cultures leading them to propose that *var2csa* represents a “default” state in the switching pattern [31].

The DC-J parasites grown in the presence of both blasticidin and chaetocin yielded a particularly informative phenotype. Parasites exposed to both drugs grew at a similar rate as the other three cultures in the early weeks of the experiment, however Q-RT-PCR analysis of the *var* gene family from RNA collected in the fourth week of growth showed that, unlike the other cultures treated with chaetocin, *var2csa* was not significantly upregulated in this population and the *bsd* gene remained dominant (Fig 7D). Continued culture of the parasites in the presence of both drugs for another two weeks showed that the growth rate began to slow significantly, with a pronounced reduction after the fifth week of exposure (Fig 7E). The poor growth of the culture and the presence of large numbers of non-replicating or dying parasites prevented us from collecting reliable gene expression data at this time point. The poor growth of the culture was not due to combined toxicity of blasticidin and chaetocin, since a similar culture in which *bsd* expression driven by a non-*var* promoter grew robustly in the presence of both drugs, and displayed the expected upregulation of *var2csa* (S15 Fig). We hypothesize that parasites subjected to double drug pressure are induced to switch to a expressing a different *var* gene, most likely *var2csa*, due to the presence of chaetocin but such parasites cannot survive blasticidin treatment because they no longer express the resistance marker. These experiments, along with the near homogenous expression of *var2csa* in parasites treated with chaetocin or the PfSET2 dom-neg for longer periods of time (Figs 2 and 4), suggest that *var2csa* induction in response to *var*-specific chromatin alterations are indeed true switching events.

### Pulsing experiments with chaetocin also demonstrate disruption of *var* switching patterns

**Fig 8:**
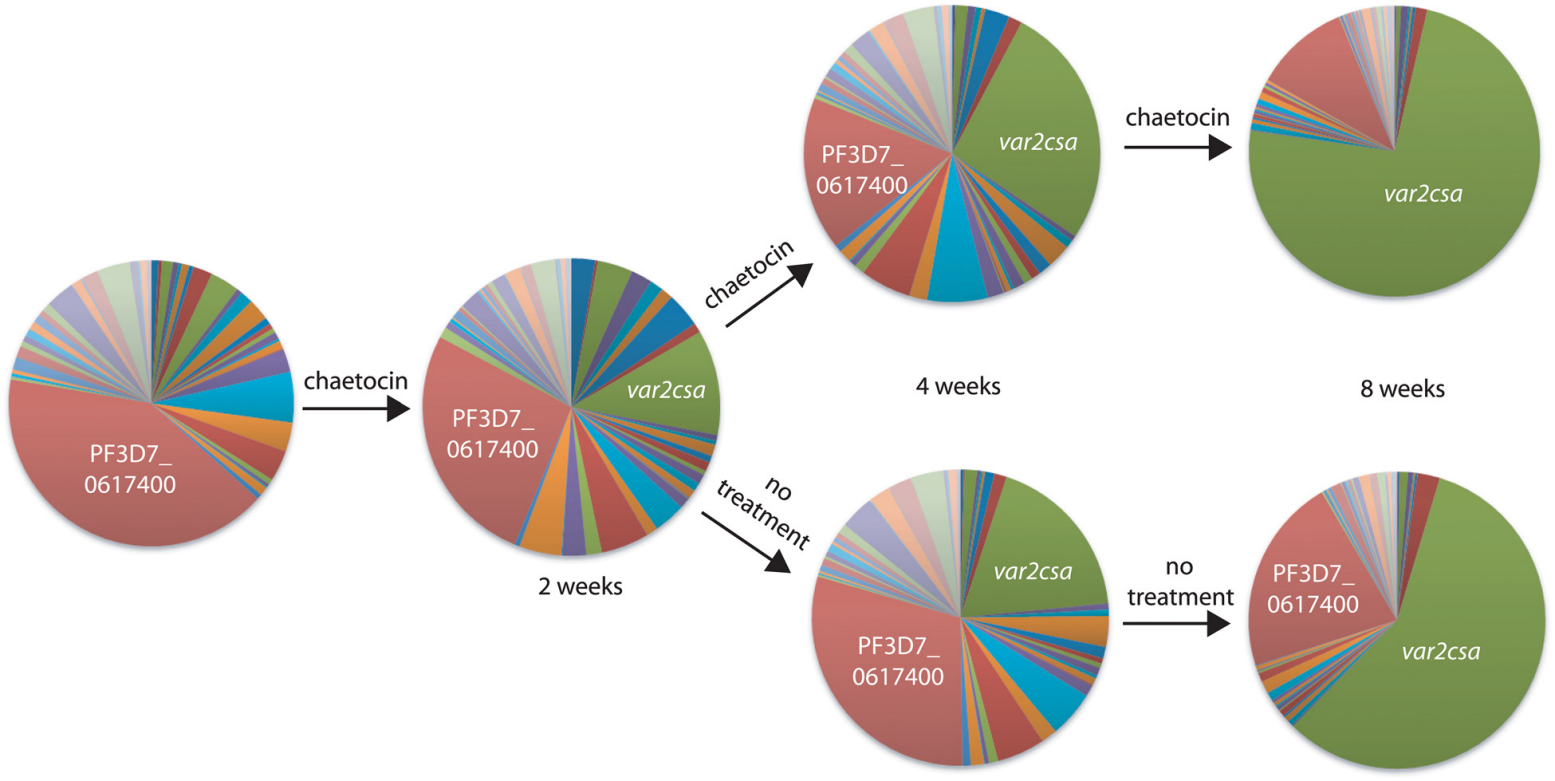
Short-term exposure to chaetocin leads to long-term induction of *var2csa* expression. Q-RT-PCR was used to determine *var* gene expression patterns and displayed as pie charts. Prior to exposure to chaetocin, the gene PF3D7_0617400 (red) was most highly expressed within this population of parasites (left most pie chart). These parasites were exposed for two weeks to chaetocin then divided into two subcultures, one which was maintained in the presence of chaetocin (top) while the other was grown in the absence of chaetocin (bottom). After 8 weeks of growth, *var2csa* (green) had become the dominant *var* transcript in both populations. Individual copy number values for each transcript are shown in S12–S14 Figs.

In the previous experiments, parasites were grown under continuous exposure to chaetocin resulting in virtually all parasites within the population switching to expression of *var2csa*. This indicates that manipulating the deposition of *var*-specific histone modifications can influence the pattern of *var* gene expression, in particular leading to the induction of *var2csa*. However it is known that epigenetic marks can influence long-term gene expression patterns, a phenomenon referred to as epigenetic memory. We were interested in how short-term exposure to chaetocin would affect longer-term *var* gene expression patterns. To address this question, we conducted “pulsing” experiments in which cultures were subjected to chaetocin treatment for two weeks as outlined previously, then the *var* switching pattern was assayed at two and six weeks after chaetocin removal. A representative example is shown in Fig 8. We observed that even after the removal of chaetocin from the culture media, induction of *var2csa* continued, though at a somewhat slower rate than observed in cultures that were exposed to chaetocin throughout the duration of the experiment. These results confirm that altering the deposition of epigenetic marks at *var* genes, even temporarily, can disrupt the long-term switching pattern of the *var* gene family.

## Discussion

While computational analyses of the *P*. *falciparum* genome reveal a relative under-representation of sequence-specific transcription factors, the epigenetic marks that modify the chromatin structure of model eukaryotes are also found within the *Plasmodium* genus. Epigenetic regulation in *P*. *falciparum* has been demonstrated to directly impact both basic biological processes as well as many aspects of host-parasite interactions that lead to disease, including antigenic variation. Of the catalog of critical histone marks found in *P*. *falciparum*, histone lysine methylation has recently emerged as one of the principal regulatory mechanisms involved in the activation, silencing, and maintenance of malaria virulence gene expression, including the *var* gene family [23,25–27]. The chromatin surrounding active genes in *P*. *falciparum*, including the single active *var* gene in the *var* repertoire, is typically tri-methylated at H3K4, which is likely catalyzed by the histone methyltransferase PfSET1 [52]. Two other histone methylation marks, H3K36me3 and H3K9me3 that are deposited by PfSET2 and PfSET3 respectively, are enriched only at telomeric regions and chromosomal regions that contain the variant antigen encoding gene families, including *var* [23,25–27]. This differs from other eukaryotic systems in which these marks tend to be distributed throughout the genome. Biochemical and genetic analyses of the histone methyltransferases of *Plasmodium* show that the enzymatic domains of the proteins are relatively well conserved and likely deposit the same marks as has been established in model eukaryotes [46]. However, outside the methyltransferase domains, the remainder of the proteins bear little similarity to their orthologs in higher eukaryotes, indicating they could display very different biological functions. For example, we previously showed that PfSET2 binds to RNA pol II CTD when it is unphosphorylated, a property distinct from its eukaryotic counterparts, and demonstrated that inhibiting this interaction has a profound effect on the *var* gene family [27].

In the current study, we utilized both genetic and chemical methods to manipulate deposition of the H3K36me3 and H3K9me3 marks that are associated with *var* gene regulation. Both over-expression of the PfSET2 dom-neg construct and treatment with sub-IC_50_ concentrations of chaetocin were designed to down-regulate, but not knockout, the methyltransferase activities of PfSET2 and PfSET3. The intent was to destabilize epigenetic regulation of the *var* gene family without completely disrupting the regulatory pathway, and thereby gain insights into how expression switching is coordinated. Surprisingly both treatments led different parasite isolates to switch expression to the unique *var2csa* gene. The preferential activation of *var2csa* in these experiments was similar, although much more rapid, compared to the observations of Mok et al who also noted frequent activation of *var2csa* in long-term cultures [31]. These studies suggest that *var2csa* occupies a unique position within the overall *var* gene regulatory hierarchy.

Since its initial discovery as the gene that encodes the form of PfEMP1 responsible for pregnancy associated malaria [43], *var2csa* has been somewhat of an enigma within the *var* gene family. While the majority of *var* genes are highly variable when compared between different geographical isolates, *var2csa* is highly conserved and seems to be found in all parasites strains [33]. This extensive conservation includes its position within the subtelomeric region of chromosome 12 and the sequence of its promoter and upstream regulatory region. This conservation extends even to the closely related parasite *P*. *reichenowi* that infects chimpanzees [33,53]. It is the only *var* gene that also displays reversible translational repression through an upstream open reading frame positioned within the 5’ leader of the mRNA, thus preventing the transcripts from being translated into protein in most instances [34,35]. While the encoded form of PfEMP1 is only thought to bind to receptors within the placenta, transcripts from the gene are also detectable in men and non-pregnant women where they are not detectably translated [36,37]. It is thought that current techniques are not sensitive enough to detect random activation of individual *var* gene without the selective advantage conferred through cytoadherence by PfEMP1. Thus the detection of *var2csa* transcripts in the absence of binding to CSA suggests a function of this gene beyond simply enabling cytoadhesion of infected cells within the placenta, and the current work suggests that it could play a role in *var* gene switching.

Recent mathematical modeling of *var* gene switching patterns, both from experimental infections and from long-term cultures, have suggested that expression of the gene family could be coordinated by a mechanism that utilizes a “switching intermediate” state [29]. In other words, parasites could switch transiently to an intermediate gene, then either rapidly switch back to the original gene, or alternatively switch to a different gene within the family. The transient activation of an intermediate gene serves as a way to prevent bias in the switching process. Without a mechanism for preventing switching bias, genes that are closely linked to the previously active gene would be expected to display a significant “position effect” and be preferentially activated. In higher eukaryotes, such position effects are well known for genes that are controlled by chromatin modification and subnuclear localization, similar to *var* genes [54,55]. However, not only do *var* genes not display this type of switching bias, examination of numerous switching events in cultured parasites found that switching to closely linked genes was actually prohibited [29]. Thus the mechanism that coordinates *var* gene expression does indeed appear to employ a method for preventing biased activation.

In their examination of a large database of *var* gene switching patterns, Recker and colleagues derived two mathematical models that robustly predict how *var* gene expression evolves over time within a population of parasites. More importantly, these models are much better predictors of actual expression patterns than random *var* gene switching. In their “lattice-type” model, they predicted the existence of certain *var* genes that are “switched to from many other variants”. These are referred to as “sink nodes” within the *var* gene switching network. While this model beautifully predicts *var* gene switching patterns, it was purely hypothetical and the molecular basis underlying such a switching network was entirely undefined and difficult to imagine. However our experiments showing selective activation of *var2csa* indicate that this gene displays characteristics consistent with a predicted “sink node”. The additional characteristics listed above (extensive conservation, translational repression, unique promoter type) make this an even more intriguing proposition. For example, translational repression would prevent *var2csa* from inducing an antibody response if it were transiently activated during a switching event. Alternatively, it is possible that different, competing switch pathways exist, and that chaetocin treatment or PfSET2 dom-neg over-expression perturbs these pathways, resulting in activation of a default pathway leading to *var2csa* expression. Either way, a role for *var2csa* as a switching intermediate or a default pathway would explain why our experiments with chaetocin and the PfSET2 dom-neg, which artificially induce and perhaps perpetuate a switching intermediate state, result in selective switching to *var2csa*. It is also noteworthy that while the genome of HB3 contains two functional copies of *var2csa*, only the copy located at the conserved locus on chromosome 12 is activated upon over-expression of the PfSET2 dom-neg construct or chaetocin treatment, suggesting that chromosomal position as well as the sequence of the *var2csa* gene is important for this phenotype.

It is worth noting that in addition to *var2csa*, there are two other types of conserved, “strain transcendent” *var* genes, *var1csa* and the so called type 3 *vars* [33,48]. *var1csa* is particularly interesting since it appears to be a pseudogene in most if not all isolates and thus does not encode a functional protein [48,56,57]. Nonetheless it appears to be transcriptionally active and highly conserved among geographical isolates, suggesting an important function beyond encoding PfEMP1. The type 3 *vars* have been shown to be transcribed and translated into uniquely small forms of PfEMP1 [58,59], however whether these proteins function as cytoadhesion molecules and if so, what they bind to has not been determined. These additional conserved *var* genes therefore share certain characteristics with *var2csa*. It is tempting to speculate that all of these genes could play roles in coordinating expression of the *var* gene family, perhaps by acting as network “sink nodes” or through as yet undiscovered mechanisms.

The speculation that *var2csa* could occupy a unique position within the *var* switching hierarchy is provocative and unifies several unusual aspects of this unique member of the *var* gene family. However, alternative interpretations are possible. For example, it is possible that a signaling cascade exists that enables parasites to detect the existence of a placenta, and that our treatments somehow activate the pathway, leading to the expression of *var2csa*. Alternatively, it is also possible that different *var* genes are characterized by different inherent switching propensities and that *var2csa* simply has the lowest threshold for activation. Thus, by depleting components of heterochromatin across the genome, *var2csa* is simply the first to escape silencing and switch to the active state. Nonetheless, regardless of the interpretation of the experimental data presented here, the unique nature of *var2csa* clearly extends to its transcriptional regulation.

In conclusion, we provide data showing that *var2csa* is unique in its propensity to become transcriptionally active in response to perturbation of *var* specific heterochromatin. This suggests it might occupy a unique position within the *var* gene switching hierarchy, potentially contributing to the coordination of *var* gene expression patterns. This information provides a first glimpse into how all of the individual *var* genes present within the parasite’s genome are co-regulated to enable the parasites to perpetuate a chronic infection. In addition, this is the first demonstration of a small molecule inhibitor that can disrupt *var* gene regulation, thus indicating a possible avenue of investigation for malaria intervention strategies.

## Materials and Methods

### Ethics statement

Human blood was used for the study and was purchased from the New York City Blood Center (NYBC) for use in parasite culture. Contact of blood donors was not attempted and was not necessary for the livelihood of the study. Informed consent was not required (other than NYBC in-house protocol). The identity of the donors cannot be readily attained by the researchers and was unknown throughout the study. The blood was used for research purposes only-solely for in vitro culture of *Plasmodium falciparum*- and not for transfusion into humans or animals. NYBC policy states that only surplus blood will be made available for research purposes and thus this study did not compromise blood supplies. The blood purchased from NYBC was only used as a resource for propagation of malaria parasites and no data was collected with regard to the blood itself. Therefore the inclusion of women, minorities or children is not applicable. These conditions indicate (and the NIH has concluded) that the study does not qualify as human subjects research.

### Parasite culture and transfection with the PfSET2 dom-neg

Both NF54 and HB3 parasite lines were cultivated following standard procedures at 5% hematocrit in RPMI 1640 medium, 0.5% Albumax II (Invitrogen), 0.25% sodium bicarbonate and 0.1 mg/ml gentamicin. All parasites were incubated in an atmosphere containing 5% oxygen, 5% carbon dioxide, and 90% nitrogen at 37°C. We utilized clonally stable NF54 bulk cultures generated by Frank et al. [28]. The subclones of NF54 as well as the HB3 isolate were transfected using DNA-loaded RBCs as described previously [60]. Briefly, parasites were loaded with 200 μg of plasmid DNA in incomplete cytomix and pulsed with a gene pulser (bio-rad). The dominant-negative version of PfSET2 was transfected alongside cultures with a control construct that encodes Firefly Luciferase as described previously [27]. Both dominant-negative and control parasites were initially cultivated in the presence of 2 μg/mL blasticidin for 42 days for the NF54 subclones but only for 14 days for HB3. Portions of the 2 μg/mL cultures were allocated to new flasks and grown in the presence of 10 μg/mL blasticidin for the time course experiments for the NF54 subclones. HB3 parasite lines were only grown at 2 μg/mL blasticidin.

### Immuno-detection of H3K36me3 and H3K9me3 from isolated *P*. *falciparum* histones

Histones from NF54 parasites transfected with the PfSET2 dom-neg as well as those exposed to chaetocin treatment were extracted and purified alongside histones from respective controls as described previously [27,61]. Briefly, infected red blood cells (RBCs) were lysed by saponin treatment and freed parasites were washed twice in 1X PBS. Parasite lysis was induced by addition of ice cold water to the pellet and incubated for 30 min on a rotator at 4°C. Nuclei from all parasite lines were pelleted at 10,000g for 10 min at 4°C. Supernatants were discarded and pellets were resuspended thoroughly in 0.4N H2SO4. The suspensions were incubated overnight on a rotator at 4°C. Samples were spun down for 20 min at 14,000g at 4°C and supernatants were dialyzed against H_2_O for 8 h. Samples were retrieved from dialysis cassettes and lyophilized in a SpeedVac overnight. Each dried sample was reconstituted in 100 μL ddH_2_O, protein concentrations determined using the Bradford assay, and analyzed by SDS-PAGE/Western to detect any changes in H3K36me3 and H3K9me3 quantities. Three antibodies, anti-H3K36me3 [27], anti-H3K9me3 (Upstate, Millipore), and anti-H3 (Abcam) were used for immunodetection.

### IC_50_ determination of histone modification inhibitors

IC50 values were determined for a series of five histone modification inhibitors that included inhibitors of histone methyltransferases, acetyltransferases and a deacetylase. The compounds used were: Histone methyltransferase inhibitors—BIX01294(hydrochloride hydrate) (Cayman) as a 16.7 mM stock in ddH_2_O, Chaetocin (Cayman) as a 14.4Mm stock in dimethyl sulfoxide (DMSO), and UNC0321 (Cayman) as a 11.7mM stock in methyl acetate; a histone acetyltransferase inhibitor Garcinol (Cayman) as a 2mM stock in DMSO; and a histone deacetylase inhibitor trichostain A (TSA) (Sigma) was prepared as a 10mM in methanol. Chloroquine (diphosphate salt) (Sigma) was used as a control in our study and prepared as a 2μM stock in PBS. Drug sensitivity assays were performed on cultured NF54 subclones using SYBR Green I as described previously [62]. Briefly, parasite cultures were synchronized using an alanine-HEPES solution [63] to obtain a synchronous culture of ring-stage parasites. 200μl cultures in 96-well plates with parasitemia of 0.2–0.5% and 2% haematocrit were incubated with increasing concentrations of each inhibitor in an airtight chamber for 72 h. 150μl of the each culture was then transferred to a 96-well black plate designed for fluorescent readings. Plates were frozen at -80°C for 3h and then thawed completely. 100μl SYBR Green diluted in lysis buffer [64] was added to each well and agitated in the dark at room temperature for 1 h. Plates were read by a SpectraMax Gemini fluorometer at an excitation wavelength centered at 490 nm and 530 nm. IC_50_ values were calculated using Graphpad Prism software as described previously [62].

### Parasite treatment with histone modification inhibitors

To investigate the effects of the six histone modification inhibitors on *P*. *falciparum*, we treated the NF54 subclones using sub-IC50 values obtained from the drug sensitivity assays. Parasites were initially treated with 0.4nM BIX01294, 900nM Chaetocin, 2nM UNC0321, 1μM Garcinol, 6.7nM TSA, and 0.3nM chloroquine separately, for 2 weeks to determine effects on *var* gene expression. For subsequent experiments, the NF54 subclones, HB3 and DC-J parasites lines were maintained in the presence of 900 nM chaetocin for the duration of the experiments, unless otherwise noted.

### Southern blots and diagnostic PCR

To confirm the copy number of *var2csa* in our HB3 isolate, the sequences of both *var2csa* copies described in the database were loaded into NEBcutter and NcoI and NsiI were determined to be restriction sites that would differentiate between the two alleles. Southern blots were performed according to established protocols. Briefly, genomic DNA was isolated from growing cultures of HB3 and digested using the restriction enzymes NcoI and NsiI. The digested gDNA was subjected to gel electrophoresis using 1% agarose in Tris base/Acetic Acid/EDTA (TAE) buffer followed by transfer to a high-bond nitrocellulose membrane by capillary action after alkaline denaturation. DNA sequences were detected using Amersham’s non-radioactive detection kit. The two *var2csa* genes were also differentiated by diagnostic PCR in which primer pairs were designed to amplify dissimilar regions of the two genes.

### RNA extraction and cDNA synthesis

RNA from all parasite lines was extracted from synchronized late ring stage parasites as described previously [51]. Briefly, RNA was extracted with TRiZol (Invitrogen) and purified on PureLink (Invitrogen) columns following manufacturer’s protocol. Isolated RNA was treated with Deoxyribonuclease I (DNase I) (Invitrogen) to degrade contaminating genomic DNA. cDNA was synthesized from approximately 800 ng of RNA in a total reaction volume that consisted of Superscript II RNase H reverse transcriptase (Invitrogen) as described by the manufacturer.

### Quantitative RT-PCR

We employed the Q-RT-PCR *var* primer set of Salanti et al to detect transcription from all *var* genes [43]. Sequences for specific primers for HB3 RNA detection are in Table 2. All reactions were performed in 10μl volumes using ITAQ SYBR supermix (Bio-Rad) or AceQ qPCR SYBR Green Master Mix Kit (Vazyme, USA) in a 7900HT RT-PCR System (ABI). ΔCT for each individual primer pair was determined by subtracting the individual CT value from the CT value of the control seryl-tRNA synthetase (User bulletin 2, Applied Biosystems, http://www.appliedbiosystems.com). ΔCTs were further converted to relative copy numbers with the formula 2ΔCT. All Q-RT-PCR assays were performed at least in duplicate with no apparent differences between runs. Relative copy numbers for each *var* gene were determined in Microscoft Excel and transcriptional profiles of individual genes are presented as pie graphs or as bar graphs. The *var* gene with the highest copy number was designated as the dominant gene.

**Table 2:** Primer sets used in real-time PCR assays specifically to amplify *var2csa* from HB3.

Primer set	Forward Primer	Reverse Primer
1 (73)	CCAAAATATAGCGAGCACA	CCTTCATCTTGCTCTTGTCG
2 (75)	TAGTGAACCTATTTATATTCGT	TGCTTCATTTCCGATGTTTG
3 (79)	TTTCTCAAGGATTGTGAGAGGTC	TTCATAGCTTCTAGCGCCTTTT

The numbers in parentheses are the original primer set # from Salanti et al. [43].

## Supporting Information

S1 Fig Bar graphs accompanying Fig 1B. Top—Parasites over-expressing firefly luciferase under selection with 10 μg/ml blasticidin. Bottom—Parasites over-expressing the PfSET2 Dominant-negative negative construct under selection with 10 μg/ml blasticidin. The primer pairs are number according to Salanti et al (2003), Molecular Microbiology, 49: 179–191. Primer pair 10 represents *var2csa*.
(PDF)

S2 Fig Bar graphs accompanying Fig 2A. The graphs represent various time points after induction of PfSET2 dominant-negative over-expression. From top to bottom, the time points are 2 weeks, 4 weeks, 6 weeks, 8 weeks, and 6 months, respectively.
(PDF)

S3 Fig Bar graphs accompanying Fig 2B. These parasites are over-expressing the PfSET2 dominant-negative construct under selection with 10 ug/mL blasticidin (top panel) or 20 ug/mL blasticidin (bottom panel).
(PDF)

S4 Fig Bar graphs accompanying Fig 2C. These parasites are over-expressing the luciferase for 2 weeks (top panel) or 6 months (bottom panel).
(PDF)

S5 Fig Bar graphs accompanying Fig 3. Top panel: *var* gene expression pattern in the original culture that displays a heterogenous expression profile. Middle panel: *var* expression pattern after over-expression of the PfSET2 dominant-negative construct for 4 weeks. Bottom panel: *var* expression pattern after over-expression of luciferase for 4 weeks.
(PDF)

S6 Fig Bar graphs accompanying Fig 4. *var* gene expression patterns are shown for an untreated clone of NF54 (top panel) and for the same clone treated with various inhibitors of histone modifying enzymes as specified above each graph.
(PDF)

S7 Fig Bar graph accompanying Fig 7 showing expression pattern of the original culture (left pie). *var* gene expression pattern for DCJ parasites starting culture at 5 ug/mL blasticidin. The last bar on the right represents the expression level of the *var* gene in which the coding region has been replaced by *blasticidin-S-deaminase* (*bsd*).
(PDF)

S8 Fig Bar graph accompanying Fig 7A. *var* gene expression pattern for DCJ parasites maintained in the presence of 5 ug/mL blasticidin for 4 weeks (left) and 6 weeks (right).
(PDF)

S9 Fig Bar graph accompanying Fig 7B. *var* gene expression pattern for DCJ parasites maintained in the presence of cheatocin for 4 weeks (left) and 6 weeks (right).
(PDF)

S10 Fig Bar graph accompanying Fig 7C. *var* gene expression pattern for DCJ parasites untreated for 4 weeks (left) and 6 weeks (right).
(PDF)

S11 Fig Bar graph accompanying Fig 7D. *var* gene expression pattern for DCJ parasites cultured in the presence of blasticidin and cheatocin for 4 weeks (left).
(PDF)

S12 Fig Bar graphs accompanying Fig 8. *var* gene expression pattern for a parasite population before treatment (top) and after treatment with chaetocin for two weeks (bottom).
(PDF)

S13 Fig Bar graphs accompanying Fig 8. *var* gene expression pattern for a parasites after treatment with chaetocin for four weeks (top) and eight weeks (bottom).
(PDF)

S14 Fig Bar graphs accompanying Fig 8. *var* gene expression pattern for a parasites after an initial treatment with chaetocin for two weeks followed by growth in the absence of chaetocin for two weeks (top) and six weeks (bottom).
(PDF)

S15 Fig *var* gene expression pattern for parasites grown in the presence of blasticidin and chaetocin. These parasite express the *blasticidin-S-deaminase* gene under the control of the *Pc-dhfr* promoter in a derivative of the plasmid pLN-ENR-GFP (Nkrumah et al. (2006), Nature Methods, 3: 615–621). After six weeks of co-selection, the parasites grew robustly and displayed the predicted upregulation of *var2csa*. The *var* gene expression profile is displayed as a pie chart (top) and as a bar graph (bottom).
(PDF)
