# Supplementary figures and images for: A Unique Virulence Gene Occupies a Principal Position in Immune Evasion by the Malaria Parasite Plasmodium falciparum
Source: PLoS Genet. 2015 May 19;11(5):e1005234. doi: 10.1371/journal.pgen.1005234 (PMC4437904; doi:10.1371/journal.pgen.1005234)

Figure S1

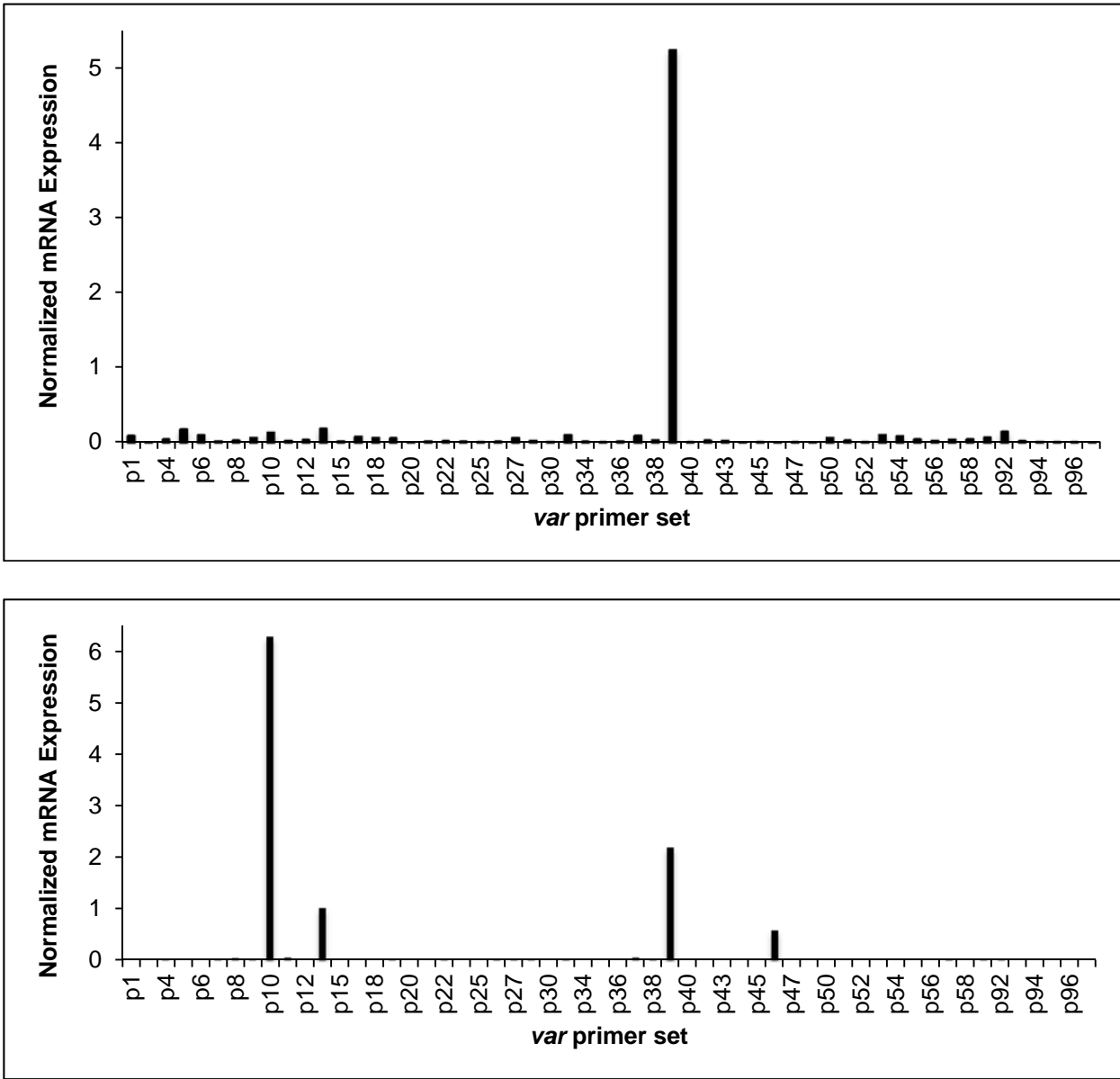

Supplement: S1 Fig — Top—Parasites over-expressing firefly luciferase under selection with 10 μg/ml blasticidin. Bottom—Parasites over-expressing the PfSET2 Dominant-negative negative construct under selection with 10 μg/ml blasticidin. The primer pairs are number according to Salanti et al (2003), Molecular Microbiology, 49: 179–191. Primer pair 10 represents var2csa. (PDF) [file pgen.1005234.s001.pdf]

Figure S2

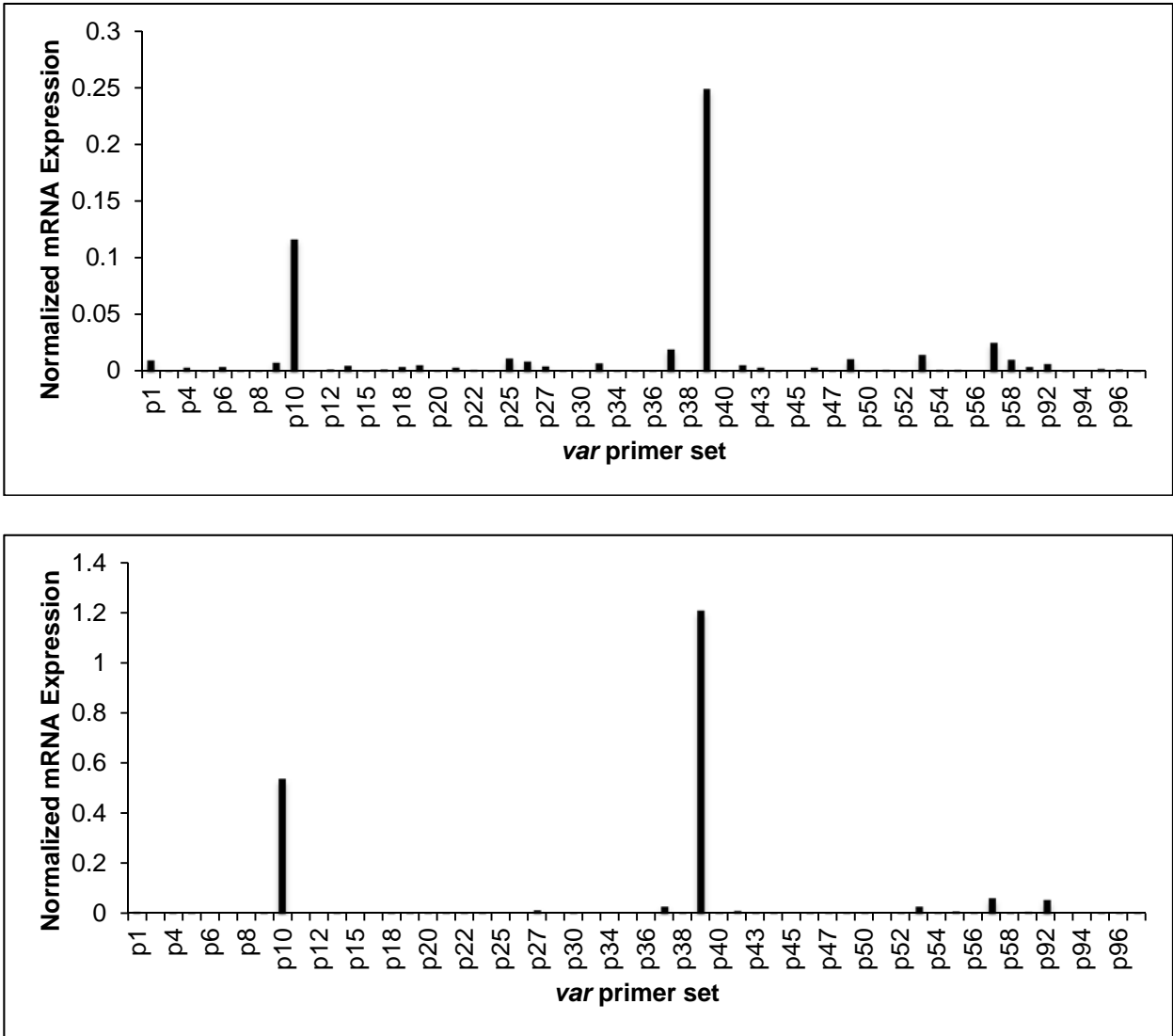

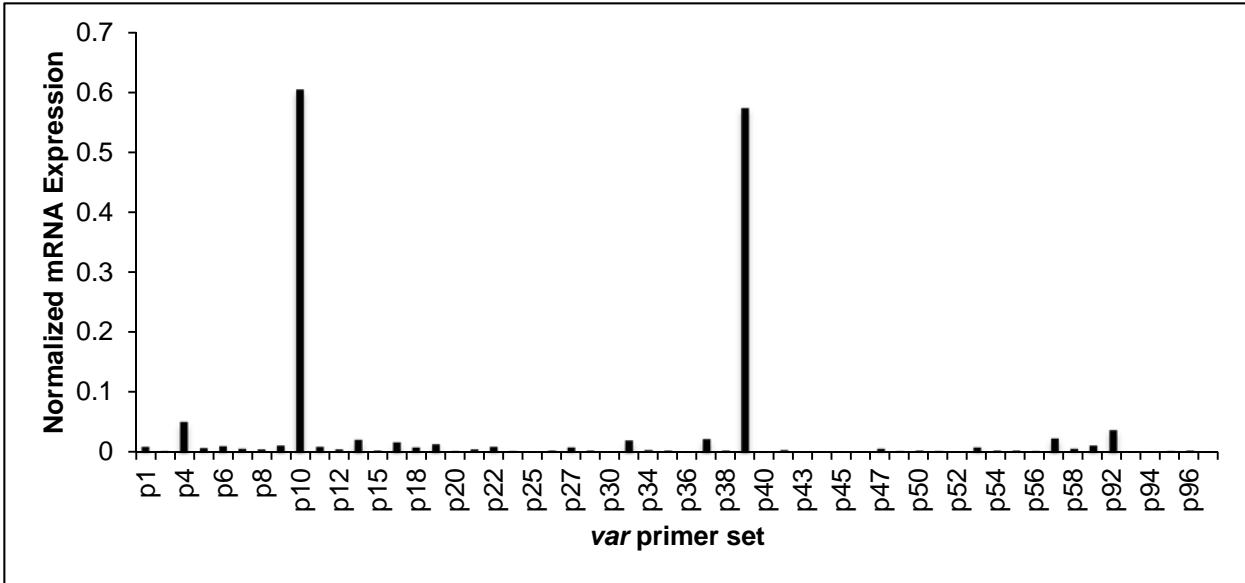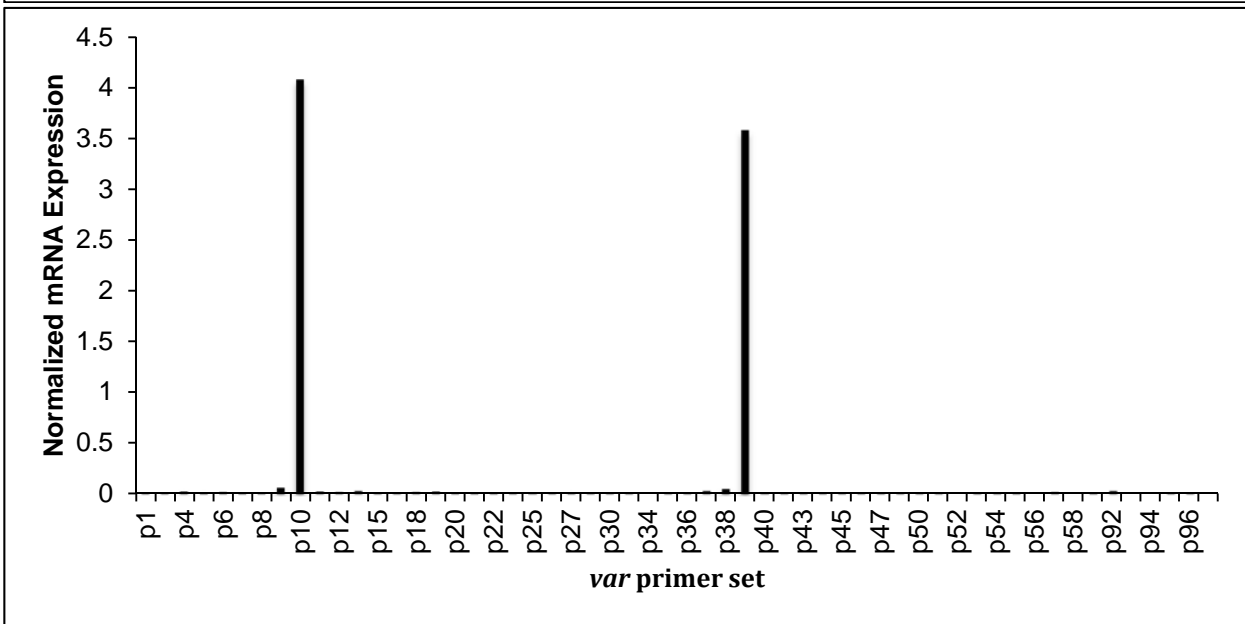

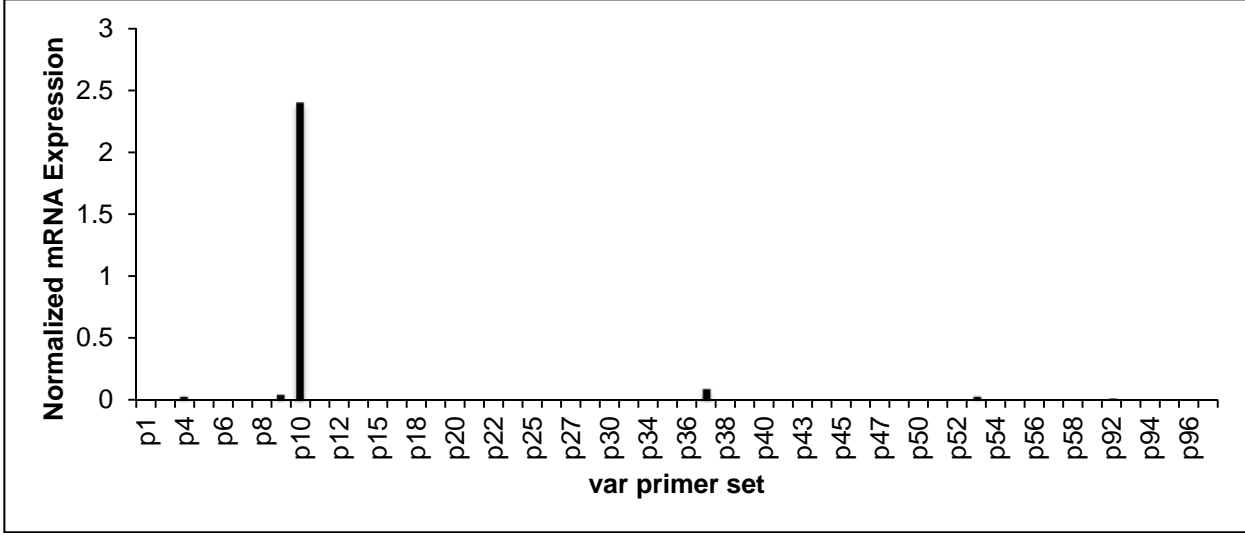

Supplement: S2 Fig — The graphs represent various time points after induction of PfSET2 dominant-negative over-expression. From top to bottom, the time points are 2 weeks, 4 weeks, 6 weeks, 8 weeks, and 6 months, respectively. (PDF) [file pgen.1005234.s002.pdf]

Figure S3

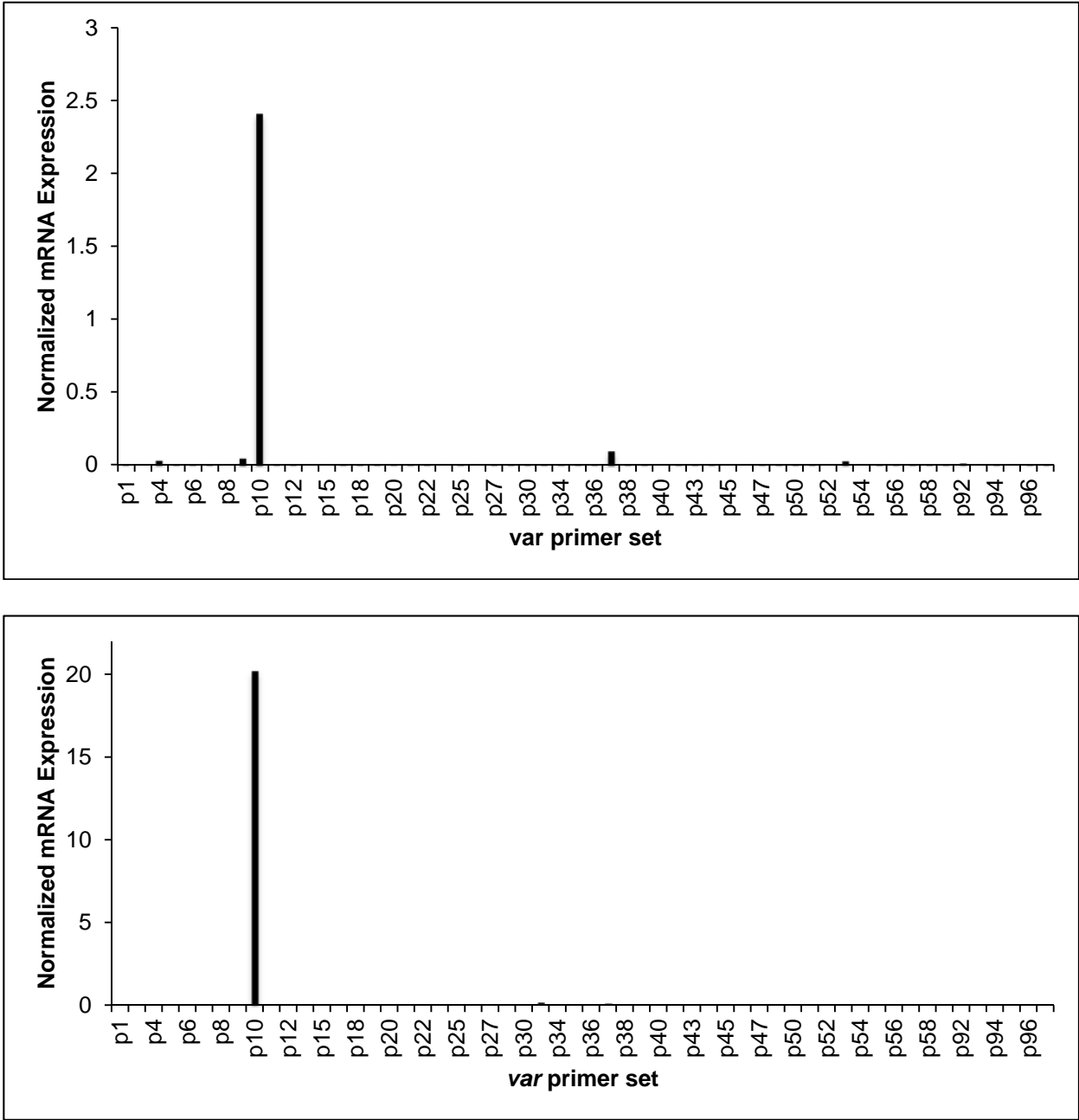

Supplement: S3 Fig — These parasites are over-expressing the PfSET2 dominant-negative construct under selection with 10 ug/mL blasticidin (top panel) or 20 ug/mL blasticidin (bottom panel). (PDF) [file pgen.1005234.s003.pdf]

Figure S4

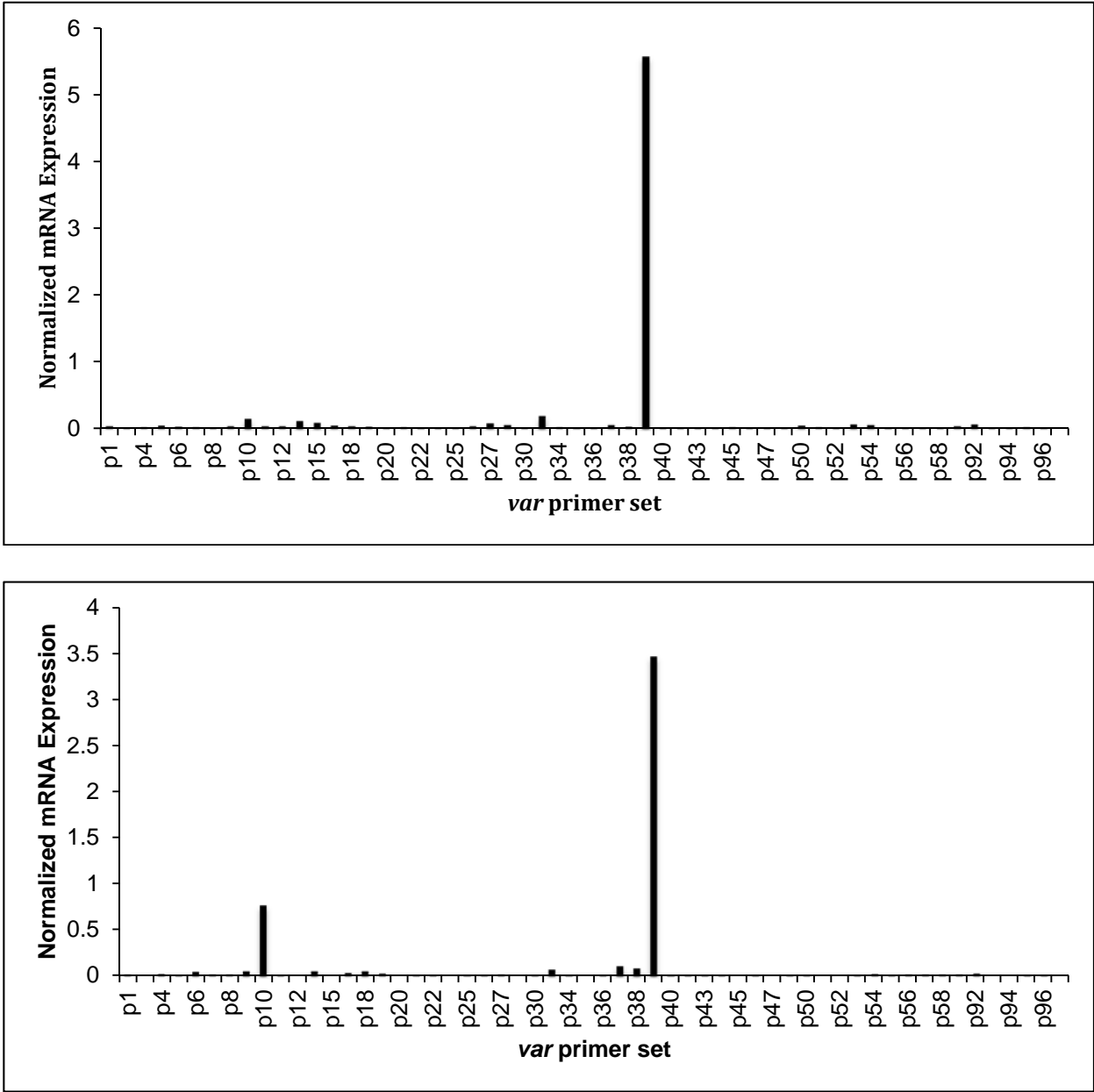

Supplement: S4 Fig — These parasites are over-expressing the luciferase for 2 weeks (top panel) or 6 months (bottom panel). (PDF) [file pgen.1005234.s004.pdf]

Figure S5

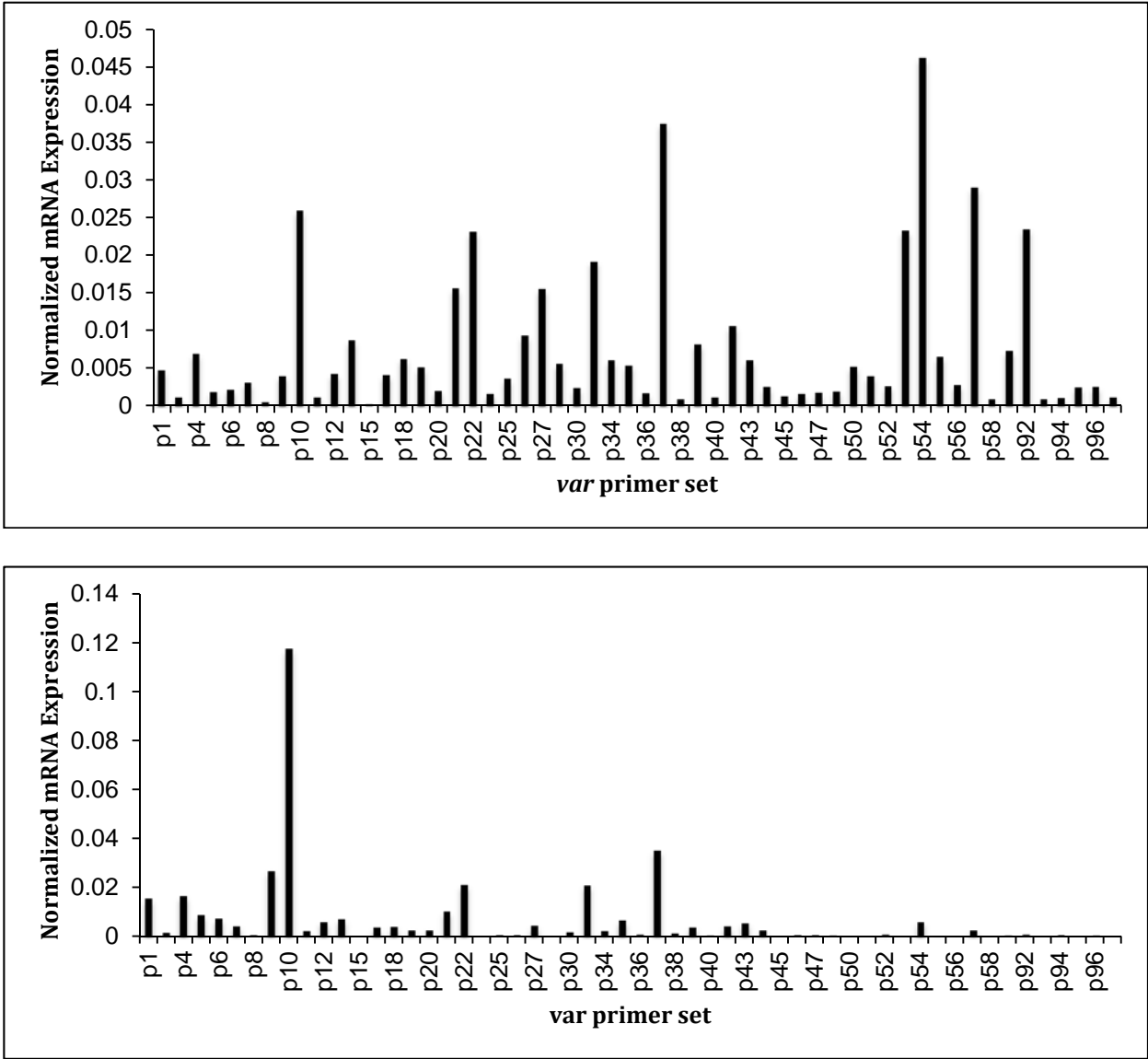

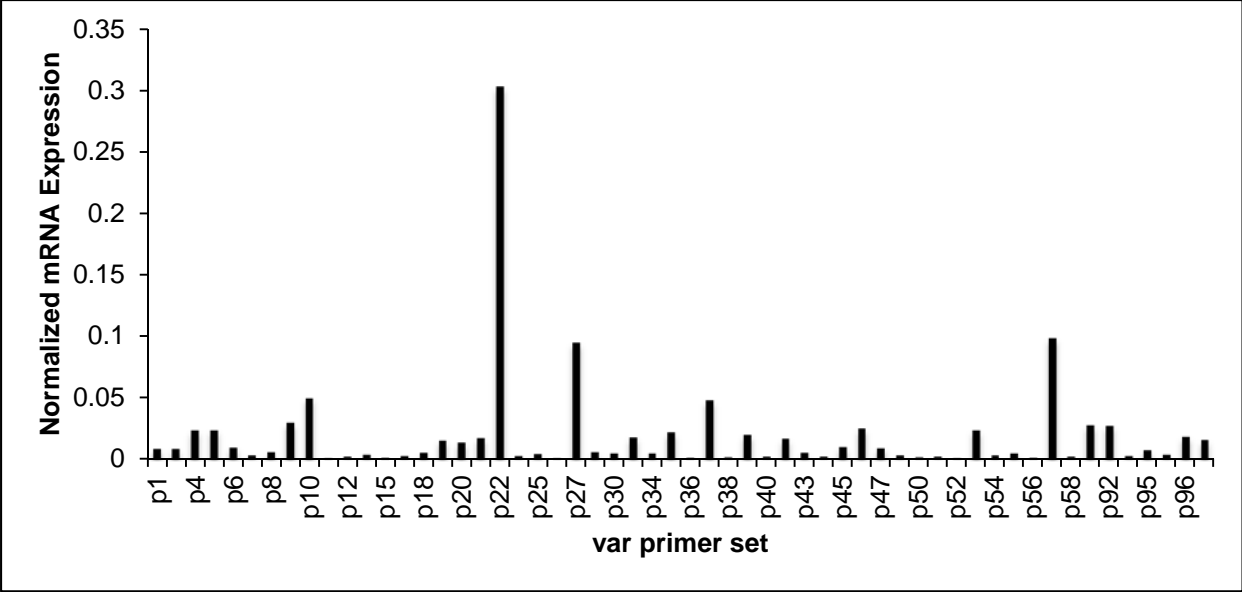

Supplement: S5 Fig — Top panel: var gene expression pattern in the original culture that displays a heterogenous expression profile. Middle panel: var expression pattern after over-expression of the PfSET2 dominant-negative construct for 4 weeks. Bottom panel: var expression pattern after over-expression of luciferase for 4 weeks. (PDF) [file pgen.1005234.s005.pdf]

Figure S6

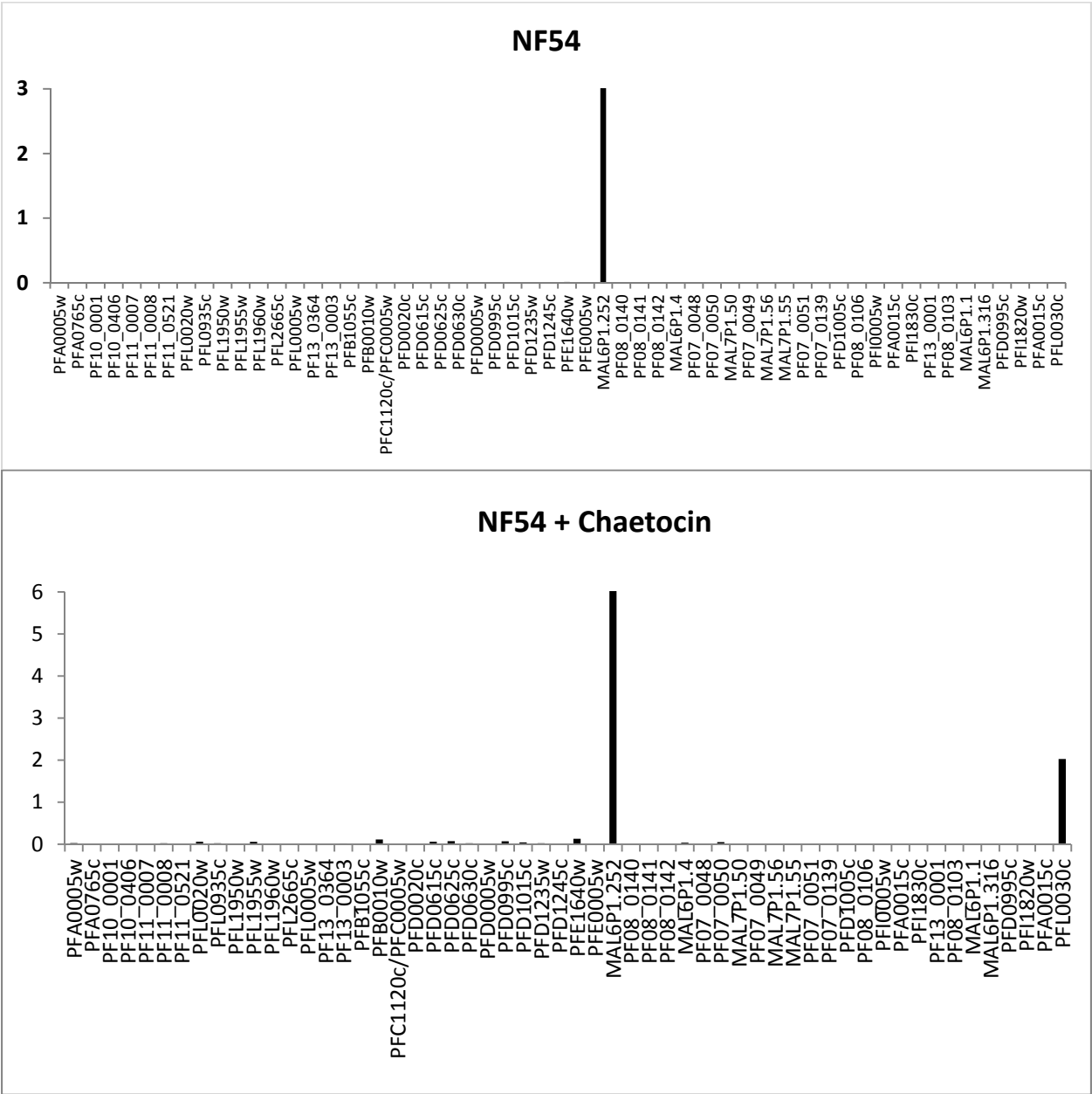

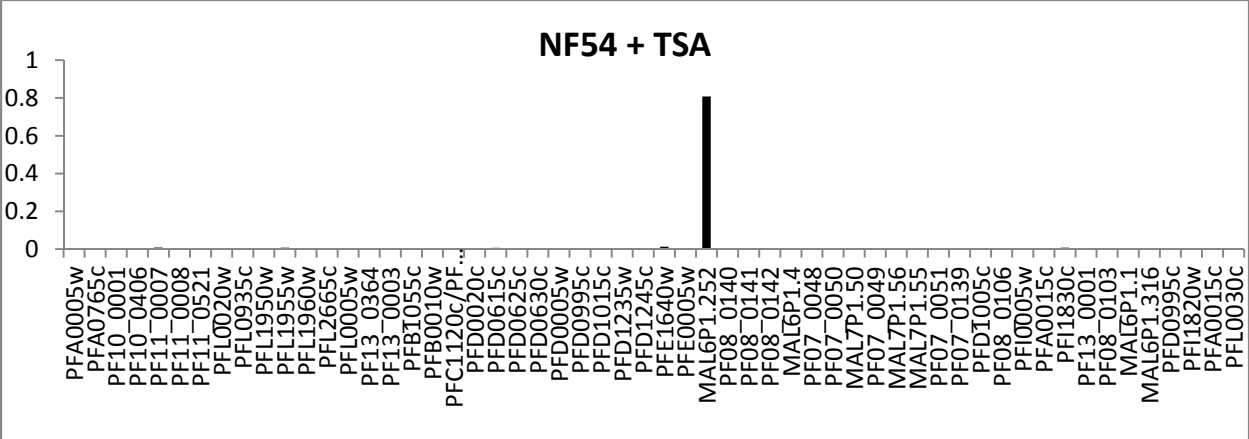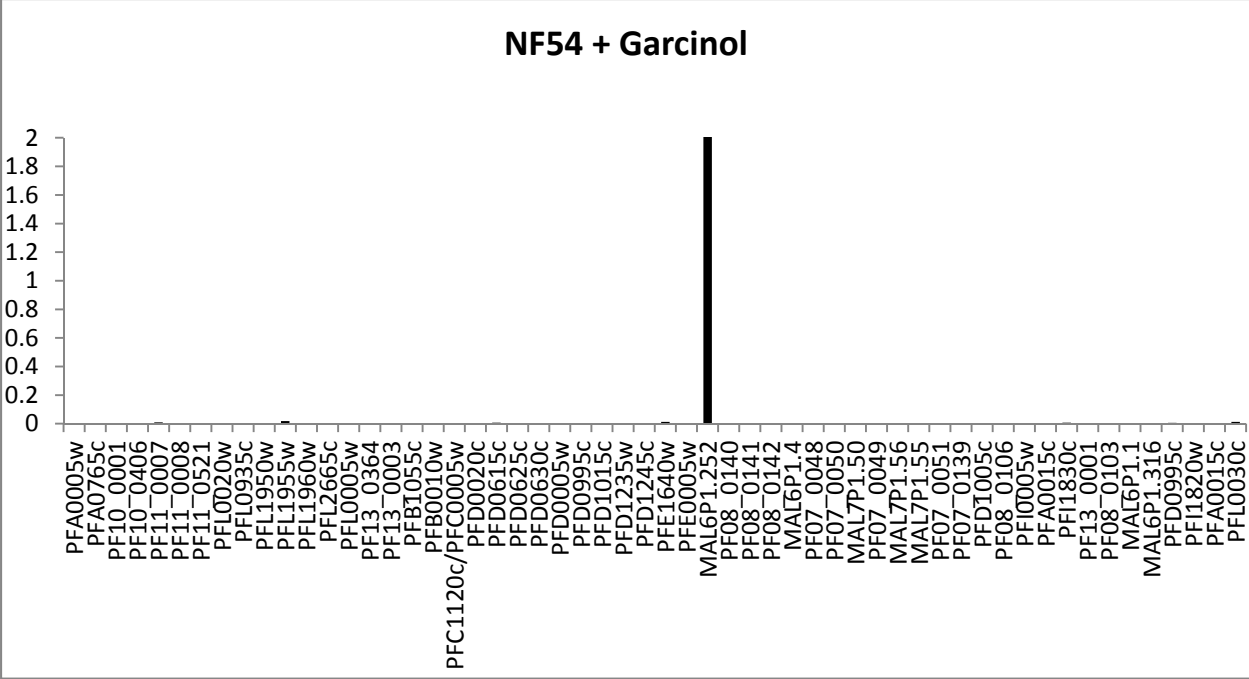

# NF54 + UNC0321

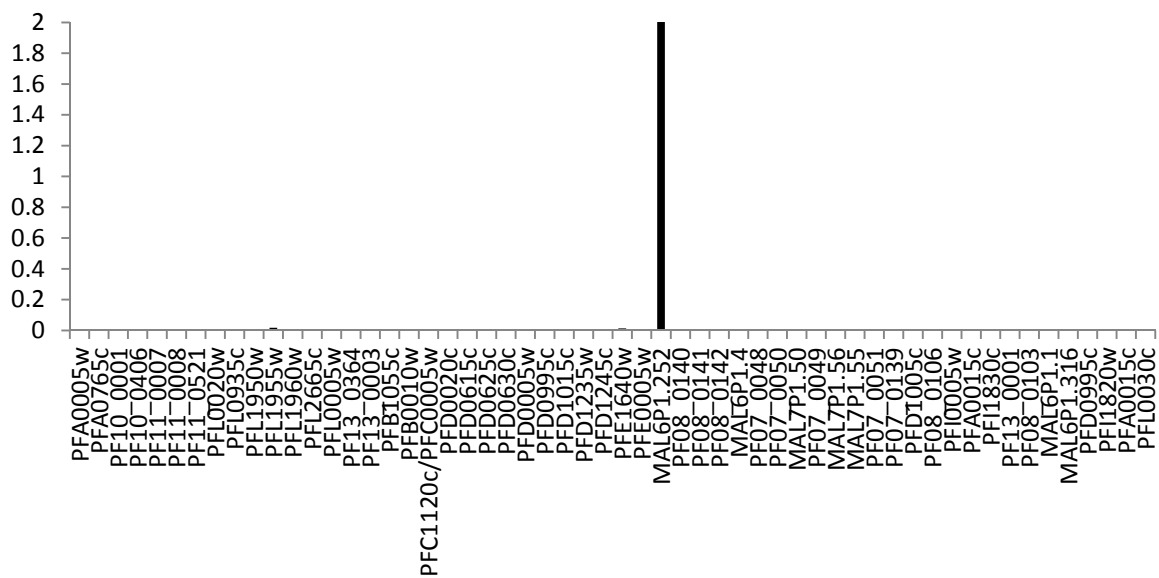

Supplement: S6 Fig — var gene expression patterns are shown for an untreated clone of NF54 (top panel) and for the same clone treated with various inhibitors of histone modifying enzymes as specified above each graph. (PDF) [file pgen.1005234.s006.pdf]

Figure S7

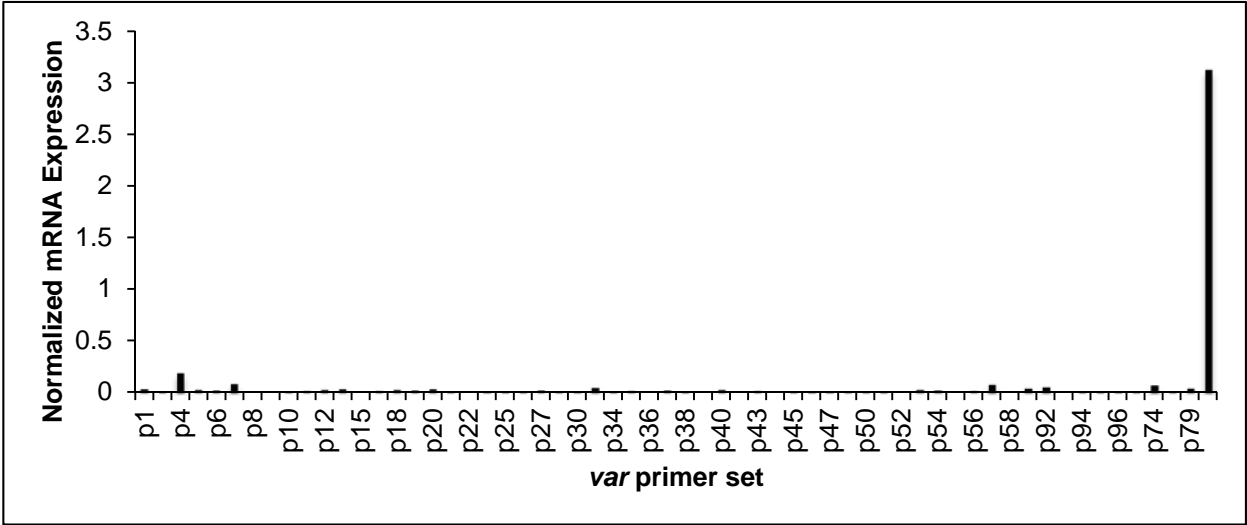

Supplement: S7 Fig — var gene expression pattern for DCJ parasites starting culture at 5 ug/mL blasticidin. The last bar on the right represents the expression level of the var gene in which the coding region has been replaced by blasticidin-S-deaminase (bsd). (PDF) [file pgen.1005234.s007.pdf]

Figure S8

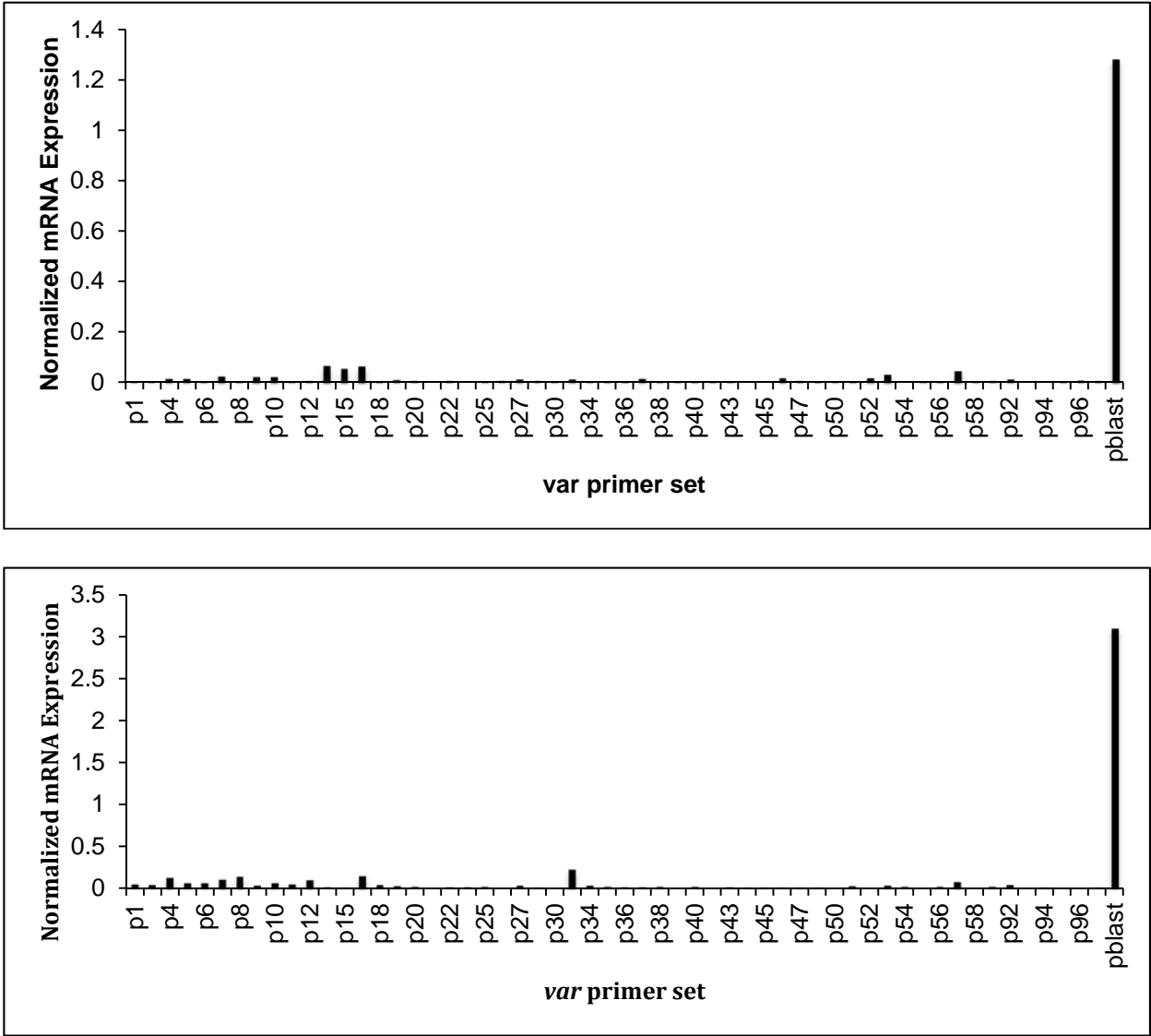

Supplement: S8 Fig — var gene expression pattern for DCJ parasites maintained in the presence of 5 ug/mL blasticidin for 4 weeks (left) and 6 weeks (right). (PDF) [file pgen.1005234.s008.pdf]

Figure S9

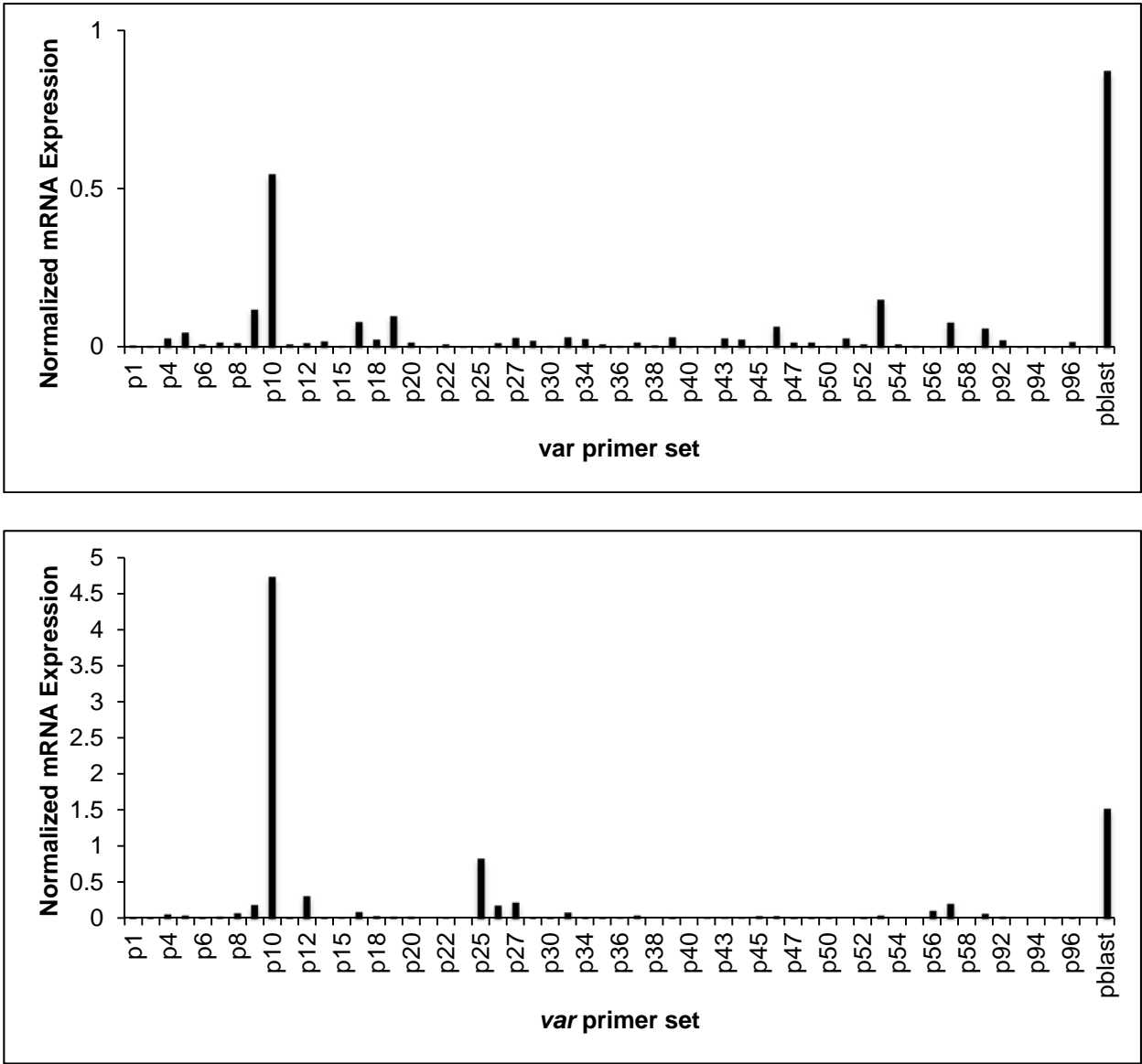

Supplement: S9 Fig — var gene expression pattern for DCJ parasites maintained in the presence of cheatocin for 4 weeks (left) and 6 weeks (right). (PDF) [file pgen.1005234.s009.pdf]

Figure S10

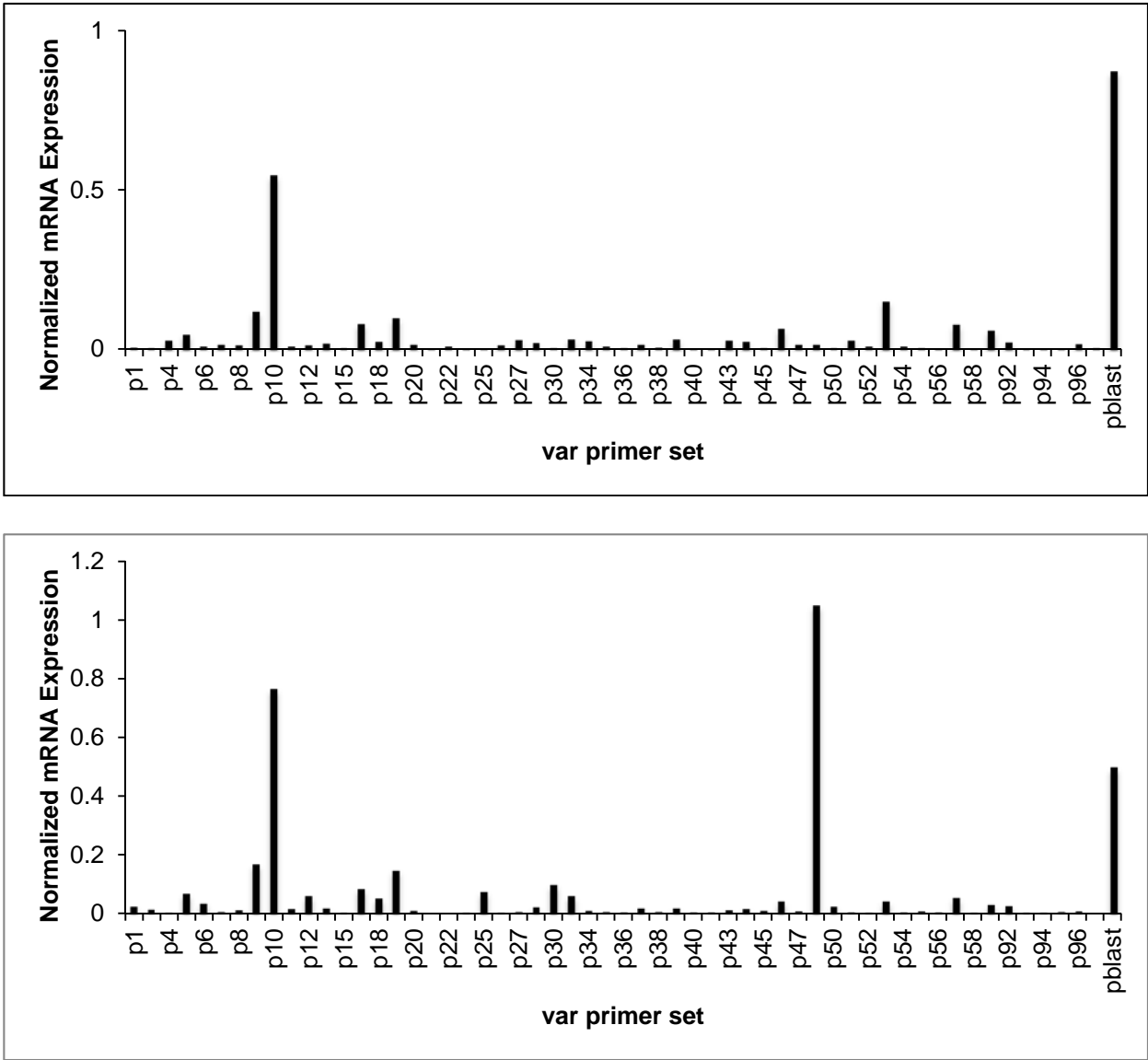

Supplement: S10 Fig — var gene expression pattern for DCJ parasites untreated for 4 weeks (left) and 6 weeks (right). (PDF) [file pgen.1005234.s010.pdf]

Figure S11

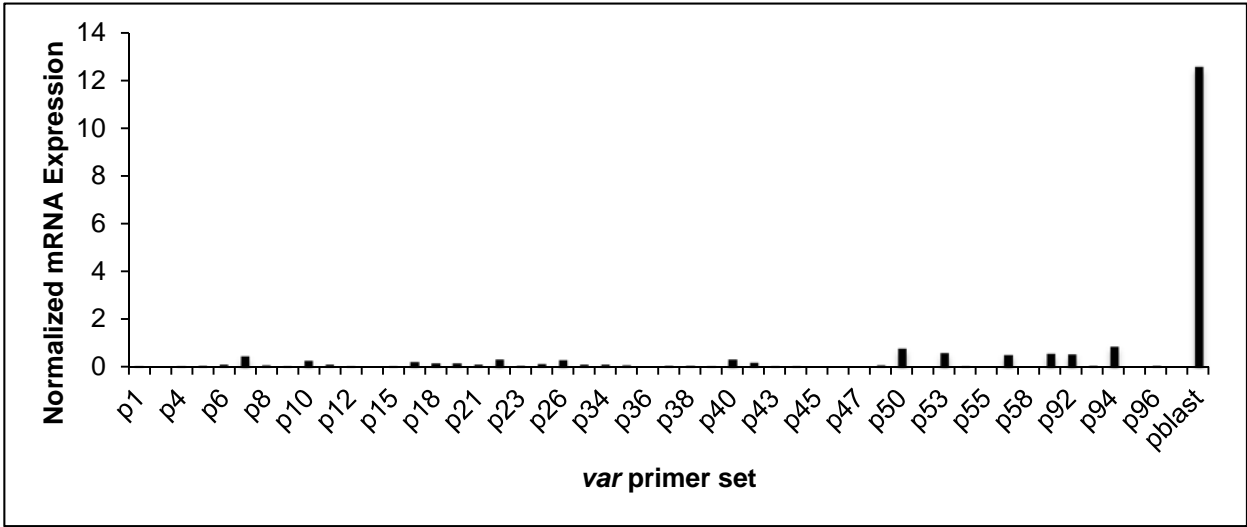

Supplement: S11 Fig — var gene expression pattern for DCJ parasites cultured in the presence of blasticidin and cheatocin for 4 weeks (left). (PDF) [file pgen.1005234.s011.pdf]

Figure S12

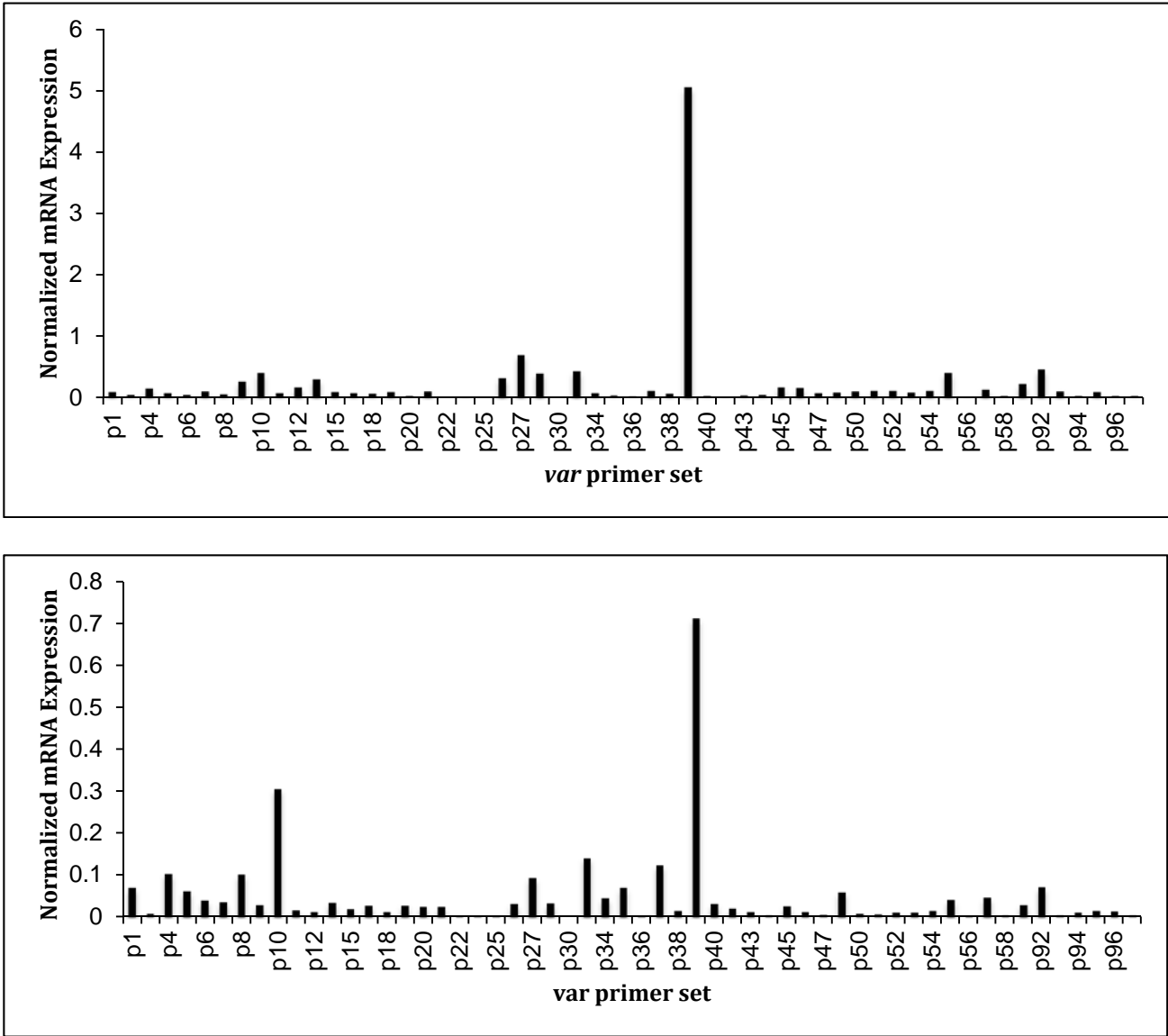

Supplement: S12 Fig — var gene expression pattern for a parasite population before treatment (top) and after treatment with chaetocin for two weeks (bottom). (PDF) [file pgen.1005234.s012.pdf]

Figure S13

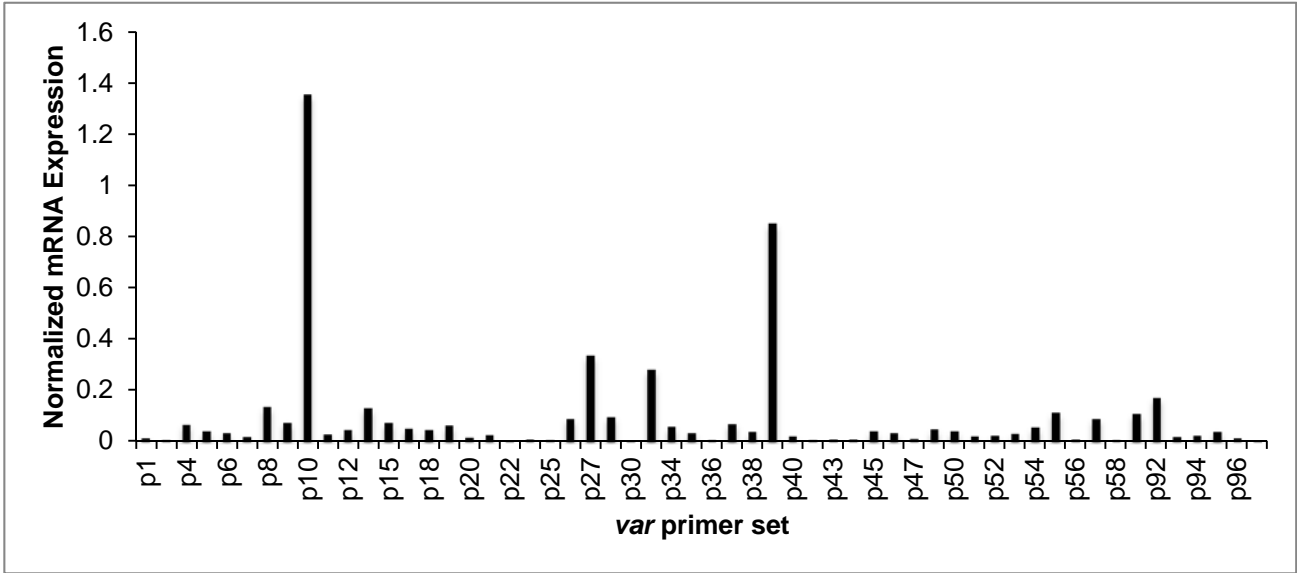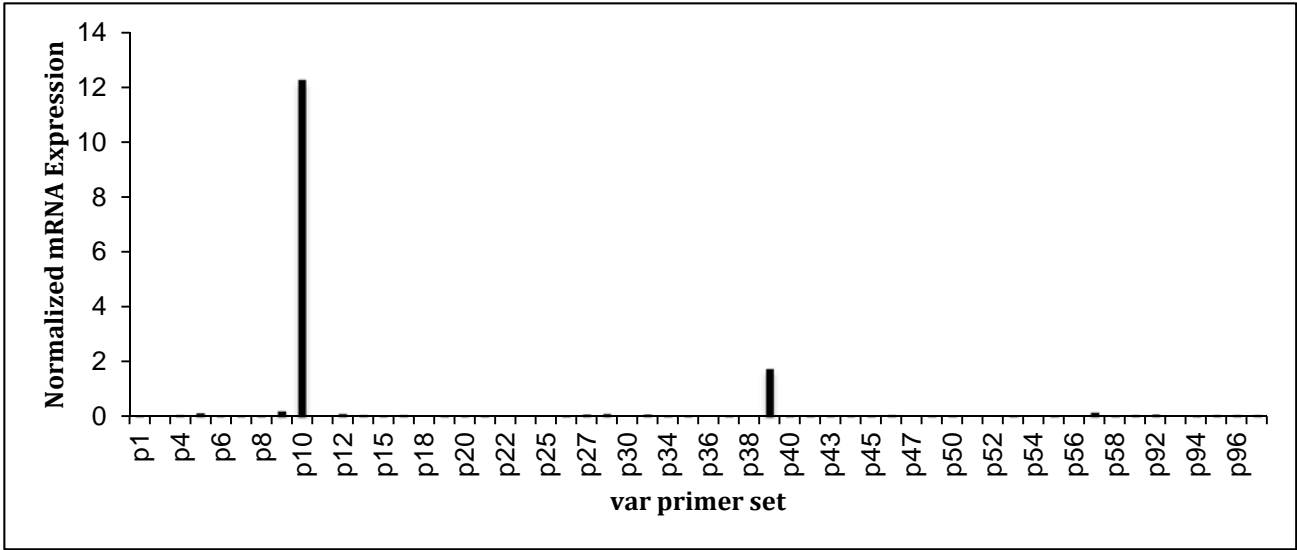

Supplement: S13 Fig — var gene expression pattern for a parasites after treatment with chaetocin for four weeks (top) and eight weeks (bottom). (PDF) [file pgen.1005234.s013.pdf]

Figure S14

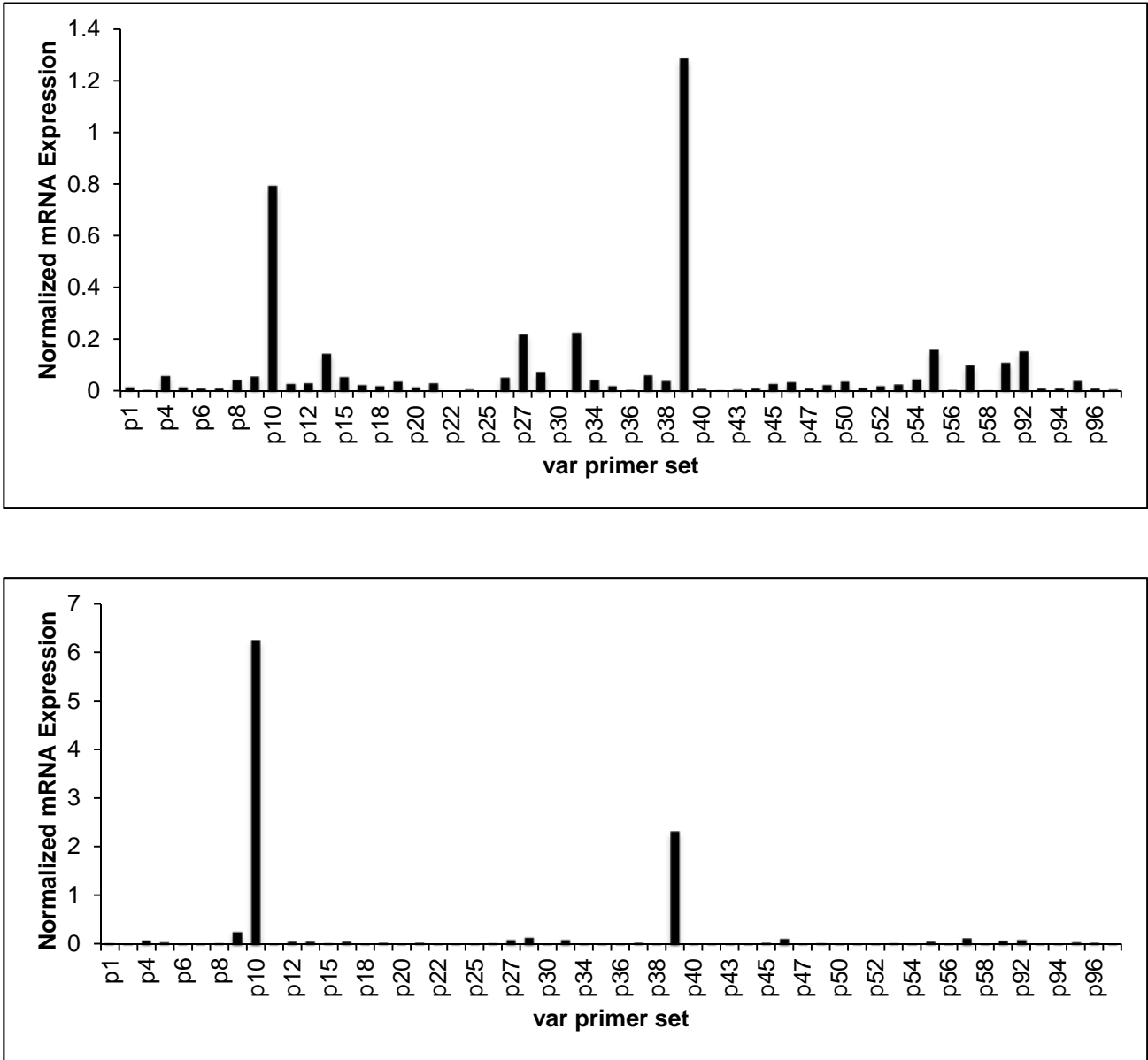

Supplement: S14 Fig — var gene expression pattern for a parasites after an initial treatment with chaetocin for two weeks followed by growth in the absence of chaetocin for two weeks (top) and six weeks (bottom). (PDF) [file pgen.1005234.s014.pdf]

Figure S15

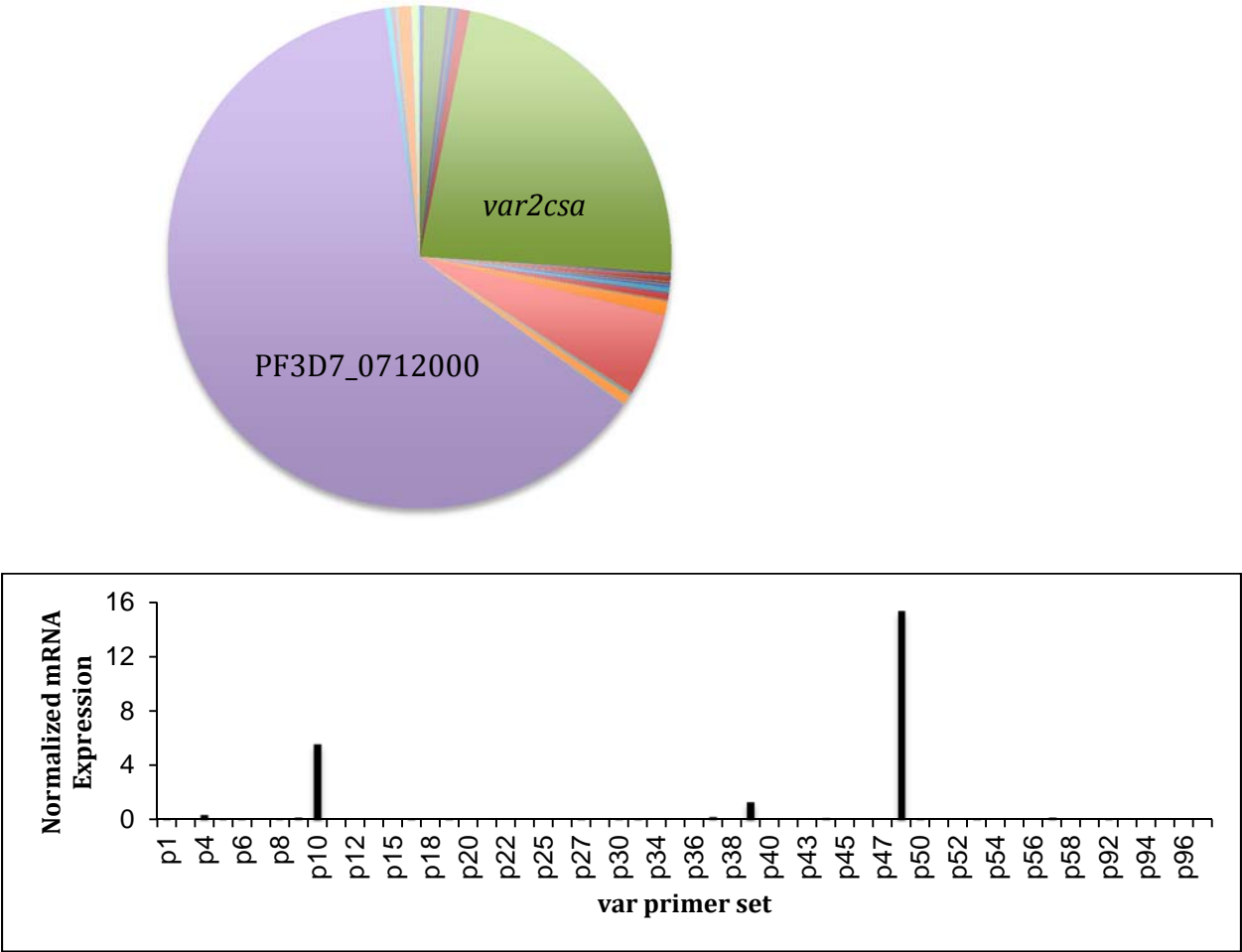

Supplement: S15 Fig — These parasite express the blasticidin-S-deaminase gene under the control of the Pc-dhfr promoter in a derivative of the plasmid pLN-ENR-GFP (Nkrumah et al. (2006), Nature Methods, 3: 615–621). After six weeks of co-selection, the parasites grew robustly and displayed the predicted upregulation of var2csa. The var gene expression profile is displayed as a pie chart (top) and as a bar graph (bottom). (PDF) [file pgen.1005234.s015.pdf]
